# Cbln1 Directs Axon Targeting by Corticospinal Neurons Specifically toward Thoraco-Lumbar Spinal Cord

**DOI:** 10.1523/JNEUROSCI.0710-22.2023

**Published:** 2023-03-15

**Authors:** Janet H.T. Song, Carolin Ruven, Payal Patel, Frances Ding, Jeffrey D. Macklis, Vibhu Sahni

**Affiliations:** ^1^Department of Stem Cell and Regenerative Biology, and Center for Brain Science, Harvard University, Cambridge, Massachusetts 02138; ^2^Burke Neurological Institute, White Plains, New York 10605; ^3^Feil Family Brain and Mind Research Institute, Weill Cornell Medicine, New York, New York 10065

**Keywords:** axon extension, cerebellin, cervical-thoracic transition, cortical neuron diversity, spinal segmental axon projection

## Abstract

Corticospinal neurons (CSN) are centrally required for skilled voluntary movement, which necessitates that they establish precise subcerebral connectivity with the brainstem and spinal cord. However, molecular controls regulating specificity of this projection targeting remain largely unknown. We previously identified that developing CSN subpopulations exhibit striking axon targeting specificity in the spinal white matter. These CSN subpopulations with segmentally distinct spinal projections are also molecularly distinct; a subset of differentially expressed genes between these distinct CSN subpopulations regulate differential axon projection targeting. Rostrolateral CSN extend axons exclusively to bulbar-cervical segments (CSN_BC-lat_), while caudomedial CSN (CSN_medial_) are more heterogeneous, with distinct, intermingled subpopulations extending axons to either bulbar-cervical or thoraco-lumbar segments. Here, we report, in male and female mice, that *Cerebellin 1* (*Cbln1*) is expressed specifically by CSN in medial, but not lateral, sensorimotor cortex. *Cbln1* shows highly dynamic temporal expression, with *Cbln1* levels in CSN highest during the period of peak axon extension toward thoraco-lumbar segments. Using gain-of-function experiments, we identify that Cbln1 is sufficient to direct thoraco-lumbar axon extension by CSN. Misexpression of Cbln1 in CSN_BC-lat_ either by *in utero* electroporation, or by postmitotic AAV-mediated gene delivery, redirects these axons past their normal bulbar-cervical targets toward thoracic segments. Further, Cbln1 overexpression in postmitotic CSN_BC-lat_ increases the number of CSN_medial_ axons that extend past cervical segments into the thoracic cord. Collectively, these results identify that Cbln1 functions as a potent molecular control over thoraco-lumbar CSN axon extension, part of an integrated network of controls over segmentally-specific CSN axon projection targeting.

**SIGNIFICANCE STATEMENT** Corticospinal neurons (CSN) exhibit remarkable diversity and precision of axonal projections to targets in the brainstem and distinct spinal segments; the molecular basis for this targeting diversity is largely unknown. CSN subpopulations projecting to distinct targets are also molecularly distinguishable. Distinct subpopulations degenerate in specific motor neuron diseases, further suggesting that intrinsic molecular differences might underlie differential vulnerability to disease. Here, we identify a novel molecular control, Cbln1, expressed by CSN extending axons to thoraco-lumbar spinal segments. Cbln1 is sufficient, but not required, for CSN axon extension toward distal spinal segments, and *Cbln1* expression is controlled by recently identified, CSN-intrinsic regulators of axon extension. Our results identify that Cbln1, together with other regulators, coordinates segmentally precise CSN axon targeting.

## Introduction

For skilled motor control, the cerebral cortex must precisely and accurately connect with specific spinal segments (Sahni et al., 2020). How corticospinal neuron (CSN) axonal projection targeting is established during development underlies motor function, CNS organization, and species differences in orofacial and forelimb dexterity during evolution. Prior work in the field has identified molecular controls over CSN specification, development, and projection targeting (Pang et al., 2000b; Arlotta et al., 2005; Chen et al., 2005, 2008; Molyneaux et al., 2005; Joshi et al., 2008; Kwan et al., 2008; Lai et al., 2008; Tomassy et al., 2010; McKenna et al., 2011; Han et al., 2011; Shim et al., 2012; Lodato et al., 2014; Muralidharan et al., 2017; Diaz et al., 2020; Sahni et al., 2021a, b).

**Figure 1.**
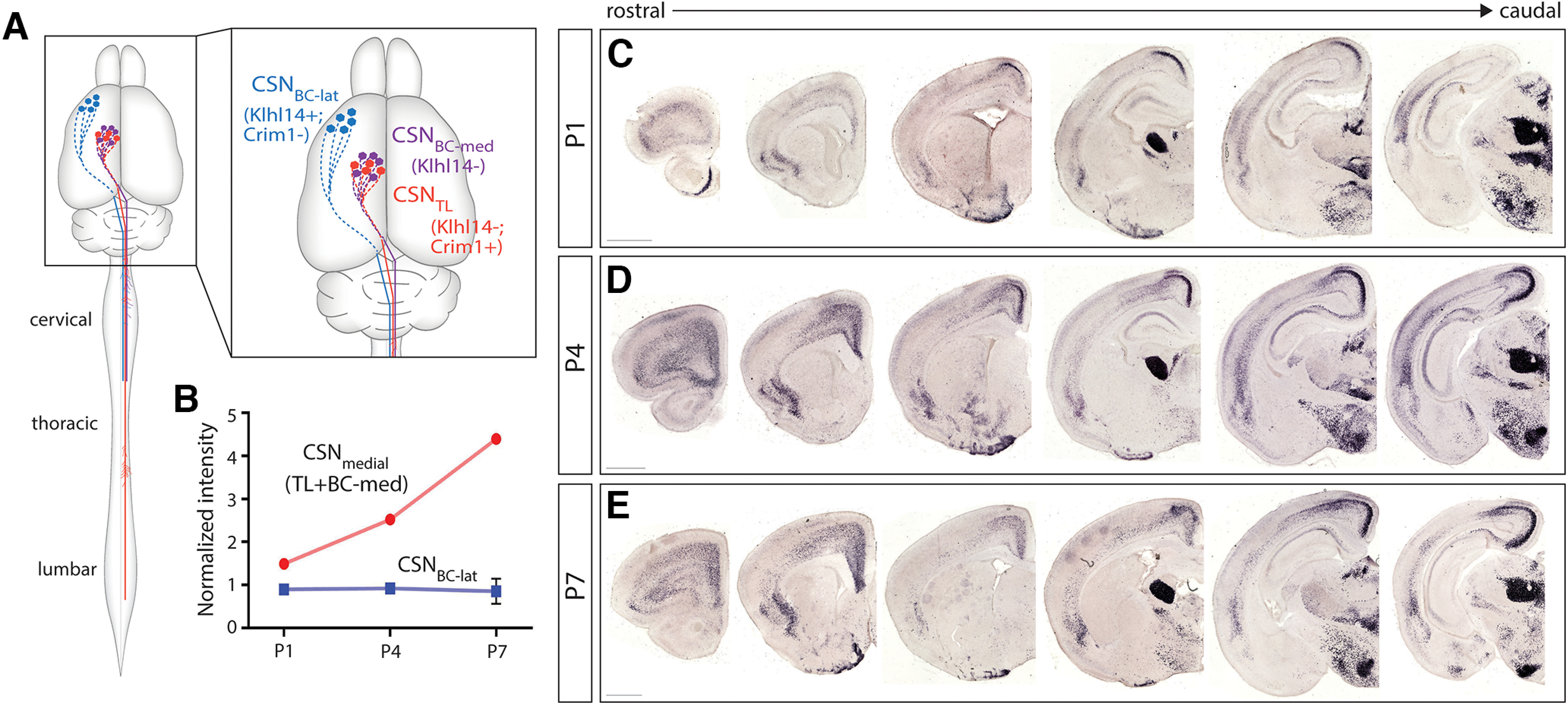
Cbln1 is specifically expressed by CSN residing in medial sensorimotor cortex. ***A***, Schematic of the mouse brain and spinal cord, with inset delineating the three spatially, segmentally, and molecularly distinct CSN subpopulations: CSN_BC-lat_ (blue) reside in rostro-lateral sensorimotor cortex and extend axons only to bulbar-cervical segments; CSN_TL_ (red) reside in medial sensorimotor cortex and extend axons to thoraco-lumbar spinal segments; and CSN_BC-med_ (purple) also reside in medial sensorimotor cortex and extend axons only to bulbar-cervical segments. CSN_TL_ and CSN_BC-med_ are both located in medial sensorimotor cortex, cannot be spatially distinguished, and are collectively referred to as CSN_medial_. *Klhl14* expression delineates *Klhl14*-positive CSN_BC-lat_ from *Klhl14*-negative CSN_medial_. Nearly all CSN_TL_ express *Crim1* while CSN_BC-lat_ are *Crim1*-negative. ***B***, Prior transcriptomic analysis comparing CSN_BC-lat_ and CSN_medial_ gene expression identified *Cbln1* as a gene that is not expressed by CSN_BC-lat_ (blue) but whose expression increases from P1 to P7 in CSN_medial_ (red; Sahni et al., 2021a). ***C–E***, *In situ* hybridization confirms that *Cbln1* is expressed in Layer V, where CSN reside. *Cbln1* expression increases from P1 to P7 and is restricted to medial Layer V throughout the rostro-caudal extent of sensorimotor cortex. Scale bars are 1 mm.

We recently identified that developing CSN subpopulations exhibit striking axon targeting specificity in the spinal white matter, and that this establishes the foundation for durable specificity of adult corticospinal circuitry. CSN_BC-lat_, which reside in rostro-lateral cortex, are relatively homogeneous, with projections to only bulbar-cervical segments. In contrast, CSN residing in medial sensorimotor cortex (CSN_medial_) are more heterogeneous, with distinct interdigitated subpopulations extending axons to either bulbar-cervical or thoraco-lumbar segments: CSN_BC-med_ extend axons only to bulbar-cervical segments, while CSN_TL_ extend axons past cervical cord to thoracic and lumbar spinal segments. We further identified that these segmentally distinct CSN subpopulations are molecularly distinct from early development, enabling molecular delineation and prospective identification even before eventual axon-targeting decisions are evident in the spinal cord: (1) *Klhl14* expression delineates *Klhl14*-positive CSN_BC-lat_ from *Klhl14*-negative CSN_BC-med_; (2) all CSN_TL_ are *Klhl14*-negative; and (3) nearly all CSN_TL_ express *Crim1* (schematized in Fig. 1*A*; Sahni et al., 2021a).

Crim1 and Klhl14 direct differential CSN axon segmental targeting by these subpopulations (Sahni et al., 2021b), indicating that the diversity of CSN axonal targeting is controlled in part by CSN-intrinsic mechanisms. Crim1 is both necessary and sufficient for CSN_TL_ axon extension to thoracic and lumbar segments. Crim1 misexpression is sufficient to redirect a subset of CSN_BC-lat_ axons into the caudal thoracic cord. However, this effect of Crim1 misexpression, though striking, only affects a minority of the overall CSN_BC-lat_ subpopulation, with the majority of CSN_BC-lat_ axons terminating in the cervical cord. In addition, although a subset of CSN_TL_ axons, which normally extend past the cervical cord, fail to extend to caudal thoraco-lumbar segments in *Crim1* null mice, ∼50% of CSN_TL_ axons still reach the lumbar cord. This indicates that some CSN_TL_ axons can extend to distal spinal targets independent of Crim1 function. Collectively, these results indicate that there are likely additional regulators that direct CSN_TL_ axon extension to distal spinal segments.

Here, we identify Cbln1 as a novel regulator of CSN axon targeting to thoraco-lumbar spinal segments. Cbln1 is a member of the C1q superfamily, which includes proteins critically essential for normal function of the immune and nervous systems (Ghai et al., 2007; Stevens et al., 2007; Yuzaki, 2011). Cbln1 has been extensively characterized in the cerebellum, where it is required for synapse formation and synapse stabilization between parallel fibers of granule cells and Purkinje cell dendrites (Hirai et al., 2005; Matsuda et al., 2010; Uemura et al., 2010; Elegheert et al., 2016; Ibata et al., 2019; Takeo et al., 2021). More recently, it also has been shown to play roles in synapse formation in the striatum and the hippocampus (Kusnoor et al., 2010; Seigneur and Südhof, 2018). There is no previously reported function for Cbln1 in corticospinal connectivity.

We find that within CSN, *Cbln1* is expressed specifically by CSN_TL_, and its expression coincides with the peak period of CSN axon extension toward thoraco-lumbar spinal segments. Misexpression of Cbln1 in CSN_BC-lat_ is sufficient to redirect axons past their normal targets in the cervical cord toward distal thoracic segments. We also identify that Cbln1 overexpression in CSN_medial_ can increase the number of axons extending past the cervical cord toward thoraco-lumbar segments. This suggests that Cbln1 is sufficient to direct thoraco-lumbar extension by CSN axons in multiple contexts. Further, this effect on CSN axon extension occurs before axon collateralization and synapse formation, establishing a novel function for Cbln1 in directing axon extension, independent of its known functions in synapse formation established elsewhere in the central nervous system. Together, these results identify Cbln1 as a novel, CSN-intrinsic determinant of thoraco-lumbar segmental axon targeting specificity.

## Materials and Methods

### Mice

CD-1 mice (Charles River Laboratories) were used for gene expression analysis, *in utero* electroporation, and AAV injections. *Fezf2* null mice were generated previously (Hirata et al., 2004) and have been described (Molyneaux et al., 2005). *Cbln1* null mice were generated and described previously (Hirai et al., 2005). Embryonic day (E)0.5 was set as the day of the vaginal plug, and post-natal day (P)0 was set as the day of birth. Mice received food and water *ad libitum*, and were housed on a 12/12 h light/dark cycle. All mouse studies were approved by the IACUC at Harvard University and at Weill Cornell Medicine. All studies were performed in accordance with institutional and federal guidelines.

### Tissue collection and preparation

Mice were anesthetized by hypothermia [postnatal day (P)0–P4] or with an intraperitoneal injection (P7–adult) of 0.015 ml/g body weight Avertin (1.25% 2-2-2 tribromoethanol in a solvent that contains 0.63% isoamyl alcohol by weight in ddH_2_O). Mice were perfused transcardially, first with PBS then with 4% paraformaldehyde (PFA) for fixation. The skull, musculature, limbs, and internal organs (viscera) were removed from the thoracic and abdominal cavities. The remaining skeletal structures were postfixed overnight in 4% PFA at 4°C. The brains were also dissected out, and postfixed overnight in 4% PFA at 4°C. The following day, the spinal cords were dissected out of the vertebral column. The brains and spinal cords were then washed in 1× PBS, and stored in 1× PBS at 4°C.

To collect embryonic tissue (E18.5), timed pregnant females were anesthetized with an intraperitoneal injection of 1 ml Avertin, and euthanized with an additional 1-ml intracardiac injection of Avertin. Embryos were dissected from the uterine horn and decapitated, and the entire head was fixed overnight in 4% PFA at 4°C. The following day, the brains were dissected out, washed in 1× PBS, and stored in 1× PBS at 4°C.

For immunocytochemistry and *in situ* hybridization, brains or spinal cords were placed in Tissue-Tek OCT Compound (Sakura Finetek) for sectioning using a cryostat (Leica CM3050 S). Before sectioning, the cerebellum, pons, and medulla were removed with a razor blade, leaving the forebrain; 50-µm coronal brain sections, or 50-µm axial or sagittal spinal cord sections, were obtained using a cryostat. All sections were stored in 1× PBS at 4°C.

### *In situ* hybridization and immunocytochemistry

Chromogenic *in situ* hybridization was performed as previously described (Arlotta et al., 2005). The primer sequences used to generate the chromogenic *in situ* hybridization probes are from the Allen Brain Atlas (http://www.brain-map.org). The *Crim1* and *Cbln1* dual fluorescence *in situ* hybridization combined with Ctip2 immunocytochemistry was performed using RNAscope probes (Advanced Cell Diagnostics; Crim1, catalog #550751; Cbln1, catalog #428551-C2) per the manufacturer's instructions. Briefly, 50-µm free floating sections were mounted on slides. Sections were then processed using the RNAscope Multiplex Fluorescent v2 kit (Advanced Cell Diagnostics; catalog #323100), with integration of the RNA protein co-detection workflow using the RNA-Protein Co-detection Ancillary kit (Advanced Cell Diagnostics; catalog #323180). The sections were treated for target retrieval followed by incubation overnight with rat anti-Ctip2 antibody, 1:100 (Abcam; catalog #ab18465; RRID:AB_2064130) at 4°C. The following day, we followed the RNAscope protocol for detecting *Crim1* (C1, Opal 520, FP1487001KT; Akoya Biosciences) and *Cbln1* (C2, Opal 650, FP1496001KT; Akoya Biosciences) gene expression followed by incubation with anti-rat Alexa Fluor 546 (Invitrogen; catalog #A-11081) for 3 h at room temperature. Sections were then coverslipped using Fluoromount-G (Southern Biotech; catalog #0100-01) for imaging.

For immunocytochemistry, brains and spinal cords were fixed and stained using standard methods (Molyneaux et al., 2005), with the primary antibody rabbit anti-GFP, 1:500 (Invitrogen; catalog #A-1112; RRID:AB_221569). For Cbln1 immunocytochemistry, we performed antigen retrieval as previously described (Kusnoor et al., 2010). Briefly, 50-µm sections were incubated in 10 mm sodium citrate buffer containing 0.05% Tween 20 at 80°C for 30 min before proceeding with primary antibody incubation. The following primary antibodies were used: rat anti-Ctip2, 1:250 (Abcam; catalog #ab28448; RRID:AB_1140055), and rabbit anti-Cbln1 (E3), 1:200 (Bao et al., 2005; Wei et al., 2007).

### Retrograde labeling of corticospinal neurons

CSN that project to lumbar spinal segments were retrogradely labeled at P5 with an Alexa Fluor 555-conjugated cholera toxin subunit B (CTB-555) recombinant retrograde tracer (Invitrogen). For these injections, mice were anesthetized under ice for 4 min, then visualized by Vevo 770 ultrasound backscatter microscopy (VisualSonics) using Aquasonic 100 ultrasound gel (Parker Laboratories). Four slow-pulse injections of 60 nl of CTB-555 (2 mg/ml) were deposited on each side of the midline at L1–L2 using a pulled glass micropipette with a nanojector (Nanoject II, Drummond Scientific) to obtain bilateral labeling. The mice were placed on a heating pad for recovery. Mice were euthanized at P7, allowing the retrograde tracers 2 d for transport.

### Anterograde labeling of corticospinal neurons

P28 mice were anesthetized using isofluorane anesthesia (2.5% in 100% O_2_), and injected in caudomedial cortex with the anterograde tracer biotinylated dextran amine (BDA) using the following stereotactic coordinates −1.0 mm lateral to the midline at bregma at a depth of 0.8 mm. A glass capillary micropipette was filled with a 10% solution of BDA (10,000 MW; Thermo Fisher Scientific) which was delivered into the cortex by iontophoresis using constant current conditions (8 µA; 7 s on 7 s off) for a total period of 20 min. Mice were perfused at P35. The injection site and labeled axons were visualized using DAB staining (Vector Laboratories).

### *In utero* electroporation

Surgeries were performed as previously described (Molyneaux et al., 2005; Greig et al., 2016). To generate the *Cbln1* overexpression construct, *Cbln1* cDNA was cloned 3′ to an EGFP coding sequence, which was driven by the CAG promoter, and the two ORFs were separated by the t2A linker sequence. In the control plasmid, the *Cbln1* cDNA was replaced with a STOP codon 3′ to the t2A linker sequence.

### AAV-mediated gene delivery

Constructs expressing GFP or *Cbln1* were packaged into AAV 2/1, a serotype known to be specific to neuronal expression, by the Massachusetts General Hospital Virus Core using established protocols. At P0, the appropriate viral mixture (103.5 ng of AAV, 0.05% DiI, and 0.08% Fast Green in 1× PBS) was injected at 23 nl per injection into specific cortical subregions using the same set-up described previously for *in utero* electroporation (Molyneaux et al., 2005; Greig et al., 2016). Briefly, for all intracortical AAV injections, we ensured localized injection and minimal injection into the ventricles by inserting the needle only into the cortical parenchyma under ultrasound guided backscatter microscopy. AAV particles were then injected in increments of 23 nl at a rate of 23 nl/s. This approach ensures localized intracortical injections in neonatal mice. Before analysis, we also used whole mount images of the cortex to ensure that cortical injection sites were well-matched between control and experimental mice. All viral work was approved by the Harvard Committee on Microbiologic Safety, and the Institutional Biosafety Committee at Weill Cornell Medicine; all work was conducted according to institutional guidelines.

### Imaging and quantification

4× and 10× images of brain and spinal cord sections were obtained on a ANDOR Clara DR328G camera mounted on a Nikon Eclipse 90i microscope or a Zeiss Axioimager M2 using Stereo Investigator software (MBF Biosciences); 63× confocal Z stacks of brain sections were obtained on a Leica SP8 confocal microscope. For counts of *Cbln1*+, *Crim1*+, and Ctip2+ cells in Figure 4 and counts of Cbln1+, Ctip2+ cells in Figure 5, confocal images were obtained of medial Layer V at the rostral-caudal level indicated in Figures 4*B* and 5*A*. Cells were counted using the cell counter function in ImageJ. For Figure 4*C*, percentages represent the mean ± SEM across four mice.

For all CST quantification on axial sections, 60× or 63× confocal Z stacks of the entire CST in the dorsal funiculus were obtained on either a Biorad Radiance 2100 confocal microscope, a Zeiss LSM 880 confocal microscope, or a Leica SP8 confocal microscope. Cervical, thoracic, and/or lumbar cord axial sections were imaged using identical parameters.

For counts of CSN retrogradely labeled from lumbar L1–L2, we first examined the spinal cord to confirm the matched spinal level of the retrograde tracer injection. Following this confirmation, we imaged and analyzed matched coronal sections for each of three specific rostro-caudal levels in wild-type (WT), *Cbln1* heterozygous, and *Cbln1* null mice. *Cbln1* wild-type and *Cbln1* heterozygous mice are indistinguishable in this line (Hirai et al., 2005). The labeled neurons were counted using the cell counter function in ImageJ (National Institutes of Health). In all mice, regardless of the genotype, labeled neurons were only found in the medial cortex on retrograde injection in L1–L2.

For counts of anterogradely labeled, BDA+ axons in the cervical, thoracic, and lumbar spinal cords, we first examined coronal sections of the brain to confirm matched sites of anterograde tracer injection. We then counted the number of axons present at the cervical C1–C2, thoracic T1–T2, and lumbar L1–L2 levels from 40× brightfield images of three axial sections per spinal level using the cell counter function in ImageJ.

For EGFP+ axon counts in axial sections, three axial sections were imaged at both cervical C1–C2 and thoracic T1–T2 levels per mouse, and the axon counts were averaged from three separate sections. For axon intensity measurements in axial sections, at least three axial sections were imaged for each mouse at cervical C1–C2, thoracic T1–T2, and lumbar L1–L2. Background fluorescence intensity was measured from the maximum intensity projection of each Z stack image, and subtracted from the Z stack using ImageJ, such that the intensity of parts of the section without labeled axons was zero. The dorsal funiculus was then selected as the region of interest, and the intensity was measured in each Z stack image. The top three measurements from the Z stack were averaged as the fluorescence intensity for that section. These measurements were then averaged for at least three axial sections at each spinal cord segmental level.

For axon extension experiments, the thoracic cord was sectioned sagittally, and every section that contained a labeled axon was imaged. Each such section was imaged in its entirety, from rostral to caudal and throughout the medio-lateral *z*-axis. Z stacks for each such section were collapsed to a single two-dimensional plane using the “create focused image” function on the NIS-Elements acquisition software (Nikon Instruments). The collapsed sections were then further combined into one two-dimensional image per mouse by aligning the edges of each section and performing a maximum intensity projection across all sections in Adobe Photoshop using the “Lighten” mode with 100% opacity. This single two-dimensional image per mouse was converted into a monochrome image. To quantify axon extension, we cropped the single image per mouse into dorso-ventral rectangular regions at five rostro-caudal locations (rostral-most, 25% caudal, 50% caudal, 75% caudal, and caudal-most). In each such rectangular region, the CST was then selected as the region-of-interest, and fluorescence intensity was measured in ImageJ. Background fluorescence intensity was measured at an immediately adjacent location in the image and subtracted from this measurement. Intensity at each rostro-caudal level was then normalized to the intensity at the rostral-most limit of the thoracic cord. If no labeled axons were present at the rostro-caudal limit in an individual case, the intensity was set to 0 in that case.

For all of the experiments, the experimenter analyzing the images remained blinded to the experimental conditions.

### Experimental design and statistical analysis

Data are presented as mean ± SEM, with *n* indicating the number of mice used in each group for comparison. The Student's *t* test was used to assess whether the proportion of CSN axons that reach thoracic T1–T2 from cervical C1–C2 or that reach lumbar L1–L2 from cervical C1–C2 was significantly different between mice injected with GFP (control) or Cbln1. We used the two-tailed Student's *t* test for the *in utero* electroporation experiment, then used the one-tailed Student's *t* test in the AAV injection experiments to test the hypothesis that *Cbln1* promotes axon extension into the thoracic spinal cord. To model the distribution of the proportion of axons that reach T1–T2 from cervical C1–C2 (T1/C1) following injection with either control AAV-EGFP or AAV-Cbln1, we fit a mixture model of two Gaussians using the R package *mixtools*. The two-tailed Student's *t* test was also used to compare the proportion of retrogradely-labeled neurons at distinct rostral-caudal levels when comparing WT and *Cbln1* null mice. We used a two-way ANOVA with repeated measures followed by Fisher's least significant difference *post hoc* test for the axon extension analyses. Data distribution was assumed to be normal, but this was not formally tested. Male and female mice were used without distinction in experiments.

## Results

### Cbln1 is expressed by CSN in medial, but not lateral, sensorimotor cortex during early postnatal development

We previously performed differential gene expression analysis to identify potential candidate molecular controls over CSN axon targeting to bulbar-cervical versus thoraco-lumbar spinal segments (Sahni et al., 2021a). We compared gene expression at three critical timepoints, P1, P4, and P7, between CSN in lateral sensorimotor cortex (CSN_BC-lat_), which extend projections exclusively to bulbar-cervical spinal segments, and CSN in medial sensorimotor cortex (CSN_medial_), with two interspersed and molecularly distinct subpopulations that extend projections to both bulbar-cervical (CSN_BC-med_) and thoraco-lumbar (CSN_TL_) segments (schematized in Fig. 1*A*). By P4, CSN_TL_, in contrast to CSN_BC-lat_ and CSN_BC-med_, extend axon projections toward distal spinal cord segments (Bareyre et al., 2005; Kamiyama et al., 2015; Sahni et al., 2020, 2021a). To identify candidate molecular controls that control CSN_TL_ axonal targeting specifically within CSN_medial_, we previously investigated genes that exhibit significant differential expression between CSN_BC-lat_ and CSN_medial_ at P4 (Sahni et al., 2021a, b). This work identified multiple candidate molecular regulators of axon targeting and collateralization, including *Crim1* as both a unique identifier of CSN_TL_, and a molecular control that is both necessary and sufficient to direct CSN axon extension to thoraco-lumbar segments. This further validated the original differential gene expression analyses to identify critical regulators over segmentally-specific CSN axon targeting.

Another candidate molecular control identified by this approach is *Cbln1*, which is specifically expressed by CSN_medial_ but not by CSN_BC-lat_, with peak differential expression at P4 and P7 (Fig. 1*B*). This peak coincides with the period when CSN_TL_ axons extend past the cervical cord toward thoraco-lumbar segments. This time course of significant differential expression along with known roles for Cbln1 and its family members in synaptogenesis (Hirai et al., 2005; Stevens et al., 2007; Kusnoor et al., 2010; Matsuda et al., 2010; Uemura et al., 2010; Seigneur and Südhof, 2018; Ibata et al., 2019; Takeo et al., 2021) suggested that Cbln1 might function in controlling these processes by some or all CSN_medial_.

To investigate this hypothesis, we first confirmed these transcriptomic data by investigating *Cbln1* expression in the developing sensorimotor cortex using chromogenic *in situ* hybridization. We examined expression at the three developmental times analyzed by differential gene expression analysis, P1, P4, and P7. These experiments identify that, at all three developmental times, *Cbln1* is expressed in medial, but not lateral, Layer V (Fig. 1*C–E*). *Cbln1* expression remains restricted to medial Layer V throughout the rostro-caudal extent of the sensorimotor cortex (Fig. 1*C–E*).

**Figure 2.**
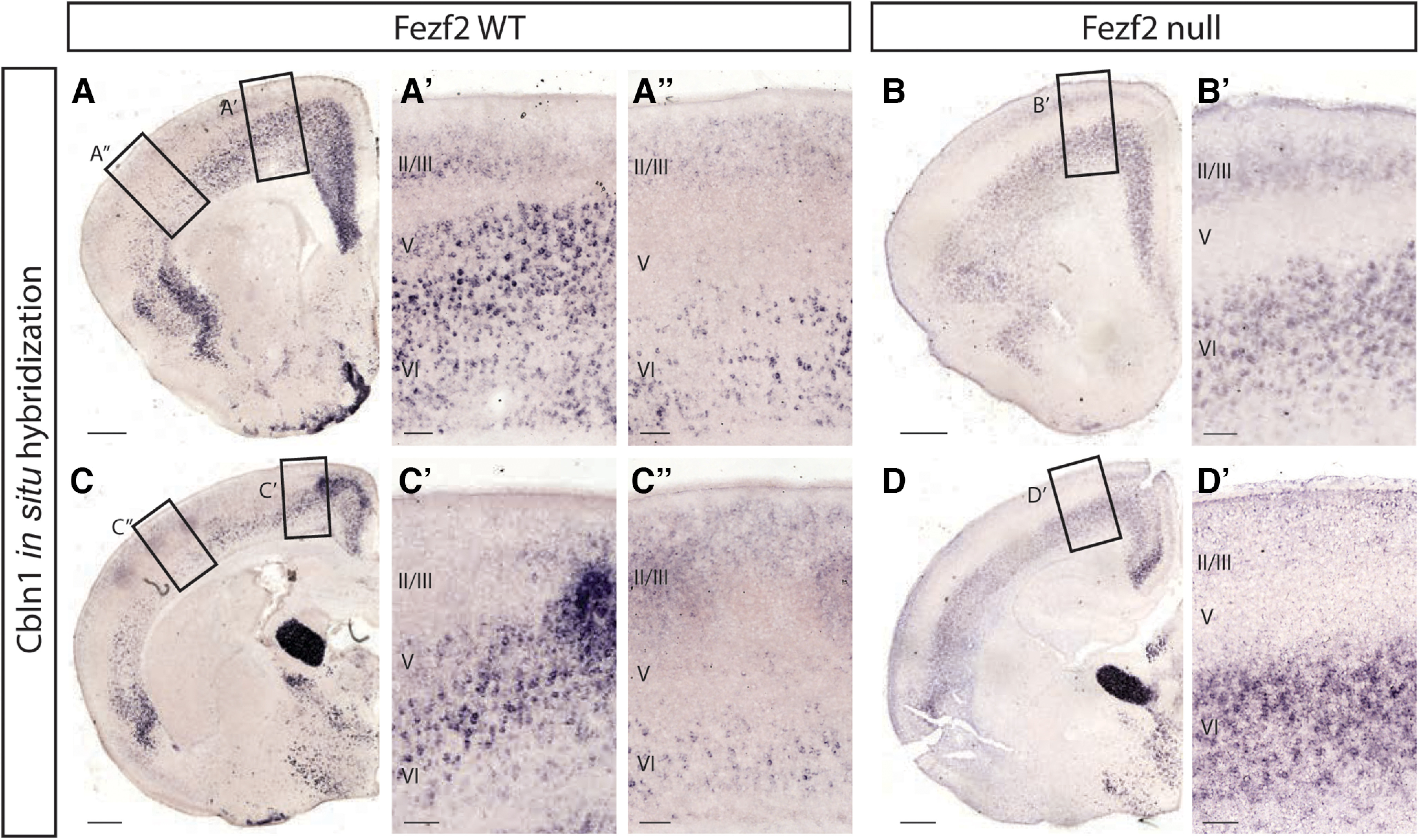
Cbln1 is expressed by CSN in Layer V. *In situ* hybridization at P7 shows that *Cbln1* is expressed in medial Layer V in *Fezf2* WT (***A***, ***C***) but not in *Fezf2* null (***B***, ***D***) mice. *Fezf2* null mice completely lack CSN (Chen et al., 2005; Molyneaux et al., 2005). This indicates that *Cbln1* is expressed by CSN in Layer V in medial sensorimotor cortex. Scale bars are 100 µm for insets and 500 µm for other images.

To confirm that *Cbln1* is expressed by CSN in Layer V, we investigated *Cbln1* expression in *Fezf2* null mice, which completely lack CSN (Chen et al., 2005; Molyneaux et al., 2005). At P7, *Cbln1* expression in Layer V is completely abolished in *Fezf2* null mice, confirming that *Cbln1* is expressed by CSN in Layer V (Fig. 2). The abolition of *Cbln1* expression in medial Layer V in *Fezf2* null mice was also observed at P1 and P4 (data not shown). As expected, *Cbln1* expression in layers II/III and VI remains unchanged in *Fezf2* null mice (Fig. 2). The expression of *Cbln1* by CSN_medial_ throughout early postnatal development suggests that Cbln1 might function in a CSN subpopulation-specific manner.

### Cbln1 is expressed in neocortical and subcortical regions during early postnatal development

**Figure 3.**
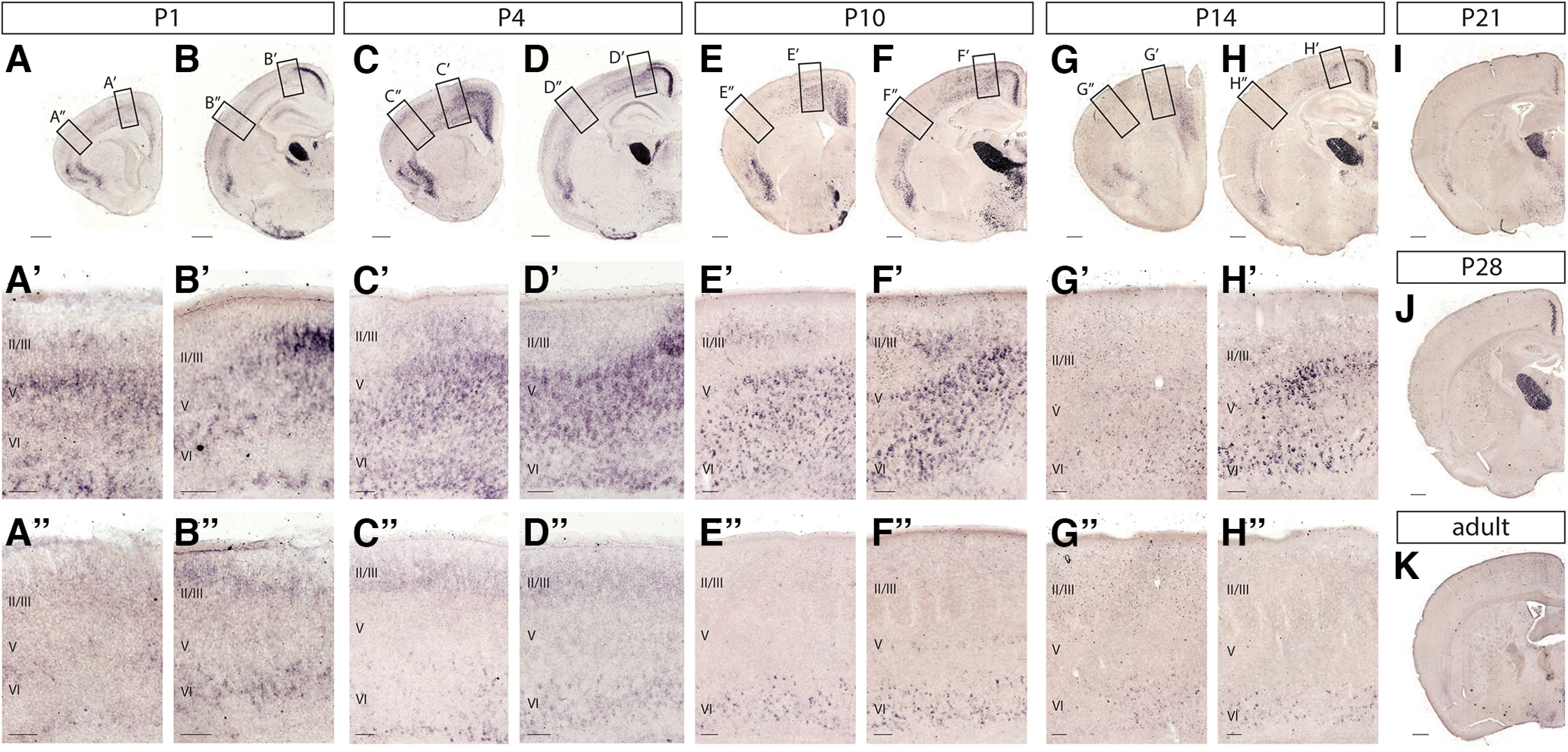
Time course of *Cbln1* expression. *Cbln1* is expressed throughout the rostro-caudal extent of sensorimotor cortex in medial but not lateral Layer V at P1 (***A***, ***B***), P4 (***C***, ***D***), P10 (***E***, ***F***), and P14 (***G***, ***H***). *Cbln1* expression in Layer V is absent in P21 (***I***), P28 (***J***), and in more than three-month-old (***K***) mice. Scale bars are 100 µm for insets and 500 µm for all other images.

To rigorously investigate the spatial and temporal course of *Cbln1* expression in sensorimotor cortex from development into maturity, we performed chromogenic *in situ* hybridization for *Cbln1* in WT mice at E18.5, P1, P4, P7, P10, P14, P21, P28, and adult (more than three months old). At E18.5, *Cbln1* is not expressed in the sensorimotor cortex (data not shown), indicating that Cbln1 is not required for early CSN development. At P1, *Cbln1* is expressed in medial Layer V throughout the rostral-caudal extent of sensorimotor cortex (Fig. 3*A*,*B*). *Cbln1* expression then steadily increases in medial Layer V from P4 to P7 (Figs. 2*A*,*C*, 3*C*,*D*). This expression decreases slightly at P10 (Fig. 3*E*,*F*). *Cbln1* is expressed at very low levels in Layer V by P14, and is absent in Layer V by P21 (Fig. 3*G–K*). *Cbln1* expression was never observed in lateral Layer V at any time point (Fig. 3). These results confirm the initial observations of differential *Cbln1* expression by CSN_medial_ versus CSN_BC-lat_ (Sahni et al., 2021a). Further, these results also highlight the temporal dynamics of *Cbln1* expression with peak expression around P7 and declining expression levels thereafter.

Within the cortex, *Cbln1* is also expressed in other layers in the neocortex and in subcerebral regions during postnatal development. In Layer VI, *Cbln1* is expressed at low levels rostrolaterally. *Cbln1* expression in Layer VI increases in caudomedial sensorimotor cortex from P1 until P10, and is present at low levels by P14 (Figs. 2, 3). *Cbln1* is expressed in medial Layers II/III, with a higher expression level in caudal versus rostral sensorimotor cortex (Fig. 3). *Cbln1* is also highly expressed in the cingulate and piriform cortex from P1 to P28, with expression decreasing with age (Fig. 3). Consistent with previous reports (Iijima et al., 2007; Kusnoor et al., 2010; Otsuka et al., 2016), we find *Cbln1* is also very highly expressed outside the neocortex, in the thalamus and the hypothalamus from E18.5 into adulthood, and in the septum at P10 and P14 (Fig. 3).

Although *Cbln1* is expressed in many neocortical and subcerebral regions, its specific restriction within Layer V to medial sensorimotor cortex combined with its distinct time course of expression suggests that Cbln1 might perform CSN_TL_-specific functions. Interestingly, *Cbln1* expression in Layer V is largely confined to early postnatal development, with expression increasing from P1 to P7 and then decreasing by P14 (Figs. 2, 3). This corresponds with the time course of CSN_TL_ axon extension to caudal spinal segments during development. CSN_TL_ first extend axons toward the lumbar cord by P5, with the number of axons reaching the lumbar cord steadily increasing from P7 to P14, with continued extension up to P28 (Bareyre et al., 2005; Kamiyama et al., 2015; Sahni et al., 2020, 2021a). The restriction of *Cbln1* expression to medial CSN with peak expression coincident with the time period of CSN_TL_ axon extension to thoracic and lumbar segments, suggests that Cbln1 might function during CSN_TL_ axon extension. Further, the temporal restriction of *Cbln1* expression in Layer V to early postnatal development suggests that the function of Cbln1 within CSN is potentially distinct from the function of Cbln1 in the cerebellum, where it is constitutively expressed throughout development and adulthood, and is required for both the proper formation and maintenance of synapses between Purkinje neurons and parallel fibers (Hirai et al., 2005).

### Within CSN_medial_, Cbln1 is specifically expressed by CSN_TL_ and not by CSN_BC-med_

**Figure 4.**
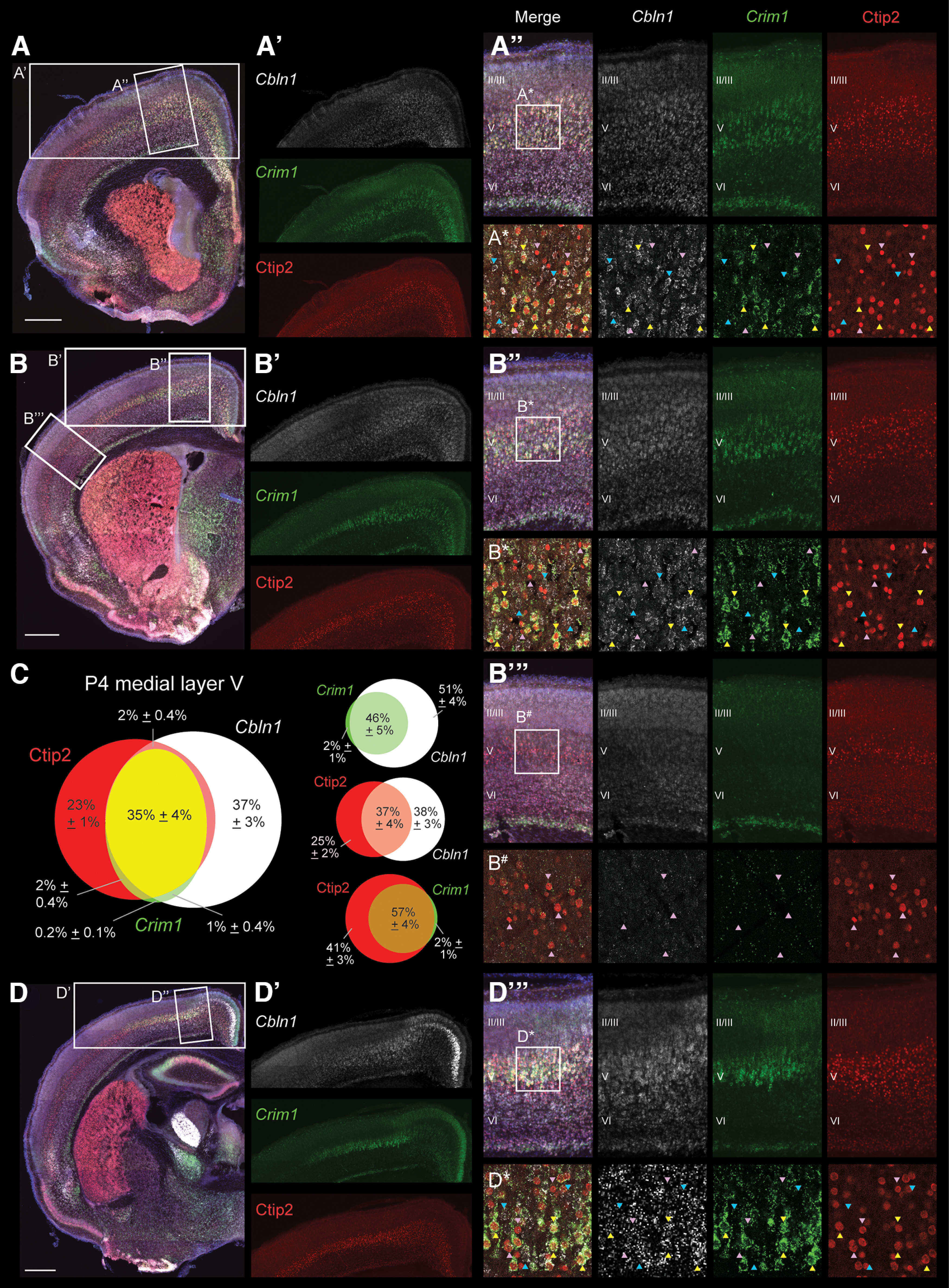
Cbln1 is expressed by CSN_TL_ and not by CSN_BC-med_. Dual fluorescence *in situ* hybridization for *Cbln1* (white) and *Crim1* (green; *Crim1* expression marks CSN_TL_) and immunocytochemistry for Ctip2 (red; high Ctip2+ marks all CSN) were performed on coronal WT brain sections at P4. Sections were also stained for DAPI (blue, nuclei). Representative sections across the rostral-caudal extent of sensorimotor cortex are shown (***A***, ***B***, ***D***). In confocal insets (A*, B*, ***B^#^***, D*), yellow arrowheads indicate examples of *Cbln1*+, *Crim1*+, high Ctip2+ cells (CSN_TL_), while blue arrowheads indicate examples of *Cbln1*+, *Crim1*−, Ctip2− cells. Finally, pink arrowheads indicate examples of *Cbln1*−, *Crim1*−, and high Ctip2+ neurons in either medial Layer V (A*, B*, D*), which correspond to CSN_BC-med_, or in lateral Layer V (***B^#^***), which correspond to CSN_BC-lat_. The overlap of *Cbln1*, *Crim1*, and Ctip2 expression was quantified in four mice in medial Layer V (***C***). We find that within CSN_medial_ (high Ctip2+), *Cbln1*+ neurons are almost exclusively *Crim1*+, indicating that *Cbln1*+ CSN are CSN_TL_. Scale bars are 500 µm.

CSN_medial_ are comprised of two interdigitated subpopulations: CSN_BC-med_, which extend axons to bulbar-cervical segments, and CSN_TL_, which extend axons past the cervical cord to thoraco-lumbar segments (Sahni et al., 2021a). We previously identified that CSN_TL_ specifically express *Crim1*, while CSN_BC-med_ are *Crim1*-negative (Sahni et al., 2021a, b). To investigate whether *Cbln1* is expressed by CSN_BC-med_, CSN_TL_, or both subpopulations, we combined fluorescence *in situ* hybridization for both *Cbln1* and *Crim1*, along with immunocytochemistry for Ctip2, which is expressed by all CSN (Arlotta et al., 2005), on coronal brain sections from WT mice at P4 (Fig. 4). As expected, we observe high Ctip2 expression throughout the rostral-caudal and medial-lateral extent of Layer V, but *Crim1* expression is restricted to medial Layer V (compare Fig. 4*B”* and *B”'*) where *Crim1* specifically labels CSN_TL_ (Sahni et al., 2021a,b). Consistent with the *Cbln1* expression observed from chromogenic *in situ* hybridization (Figs. 1–3), *Cbln1* expression in Layer V is also restricted to the medial sensorimotor cortex.

We next quantified cells that expressed *Cbln1*, *Crim1*, or Ctip2 in medial Layer V of sensorimotor cortex (Fig. 4*C*). High Ctip2+ neurons can be segregated into two subpopulations that correspond to the previously described CSN subpopulations within medial Layer V (Sahni et al., 2021a): *Crim1*-positive CSN_TL_ that comprise 58%±4% of Ctip2+ neurons and *Crim1*-negative CSN_BC-med_ that comprise the remaining 42%±4% of Ctip2+ neurons. Further, we observe that 97%±1% of *Crim1*-positive neurons are high Ctip2+, consistent with specific expression of *Crim1* by CSN_TL_ (Sahni et al., 2021a).

Surprisingly, we find two *Cbln1*-expressing populations in Layer V: a high Ctip2+ population (50%±5% of *Cbln1*+ cells) and a Ctip2-negative population (50%±5% of *Cbln1*+ cells; Fig. 4*C*). This is in contrast to the absence of *Cbln1* expression in Layer V of the *Fezf2* null cortex (which lacks all CSN) by chromogenic *in situ* hybridization (Fig. 2). The lack of *Cbln1* expression in Layer V of the *Fezf2* null cortex suggests that all *Cbln1*+ cells would be high Ctip2+, which makes the identification of a *Cbln1*+, Ctip2− population rather surprising. This could potentially indicate cell nonautonomous effects by *Cbln1*+ CSN on *Cbln1* expression in non-CSN cells; that *Cbln1* is also expressed by other, currently unidentified cells that require *Fezf2* expression in Layer V, such as *Fezf2*-expressing callosal projection neuron (CPN; Tantirigama et al., 2014, 2016); or that *Cbln1* expression in non-CSN cells is below the detection limit of chromogenic *in situ* hybridization. Nevertheless, we find that 94%±1% of the *Cbln1*+, Ctip2+ neurons (*Cbln1*-expressing CSN) are also *Crim1*+, and 95%±2% of the *Crim1*+, Ctip2+ neurons (CSN_TL_) are also *Cbln1*+ (Fig. 4*C*). This indicates that although *Cbln1* expression in medial Layer V is not restricted to CSN, *Cbln1* expression within CSN_medial_ is almost entirely restricted to *Crim1*+ CSN_TL_ and excluded from CSN_BC-med_.

### Cbln1 protein is present in CSN_TL_

**Figure 5.**
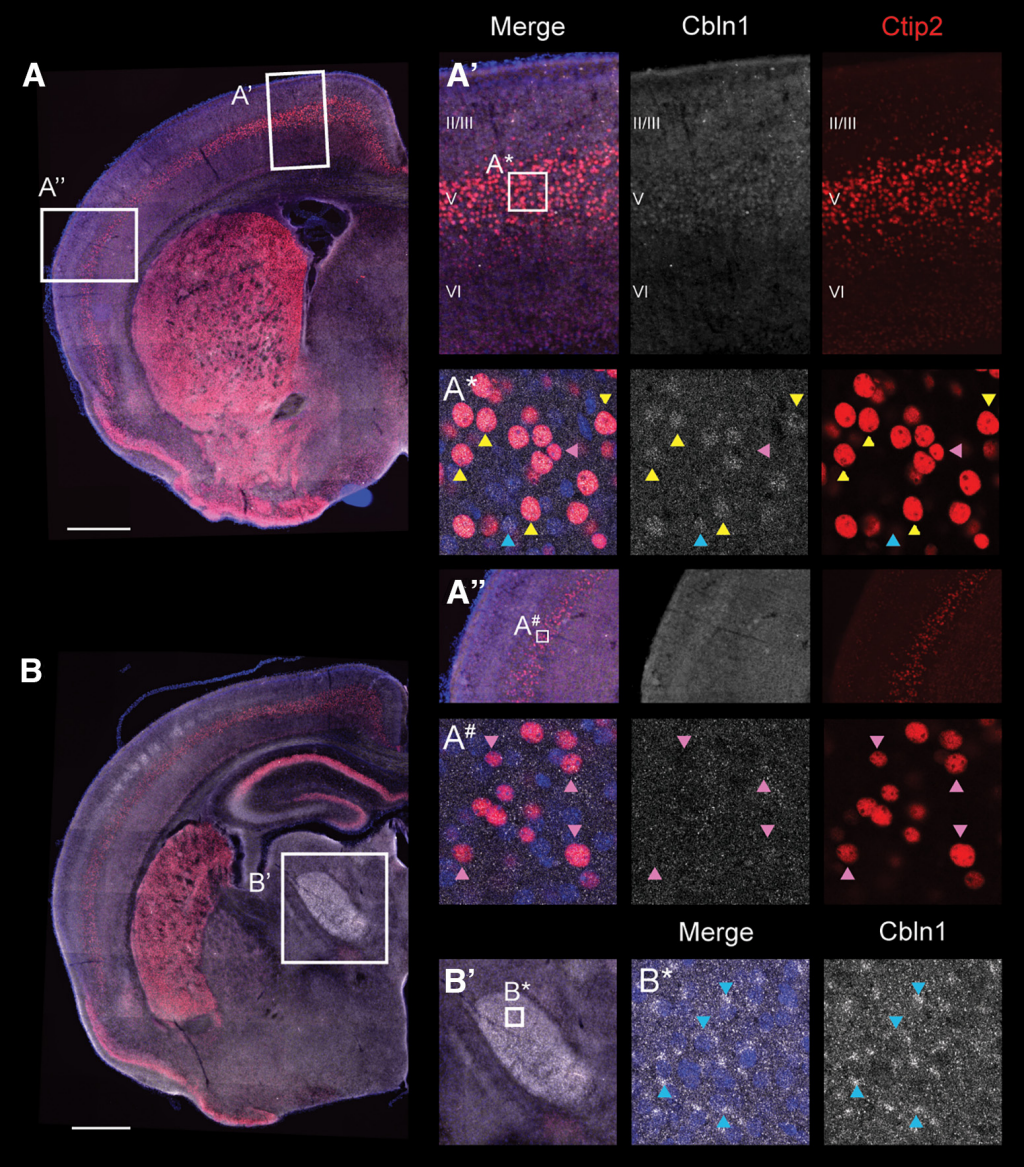
Cbln1 protein is present in CSN. (***A***) Cbln1 protein is detected in medial (***A′***) but not lateral (***A″***′) Layer V, and co-localizes with high Ctip2+ neurons (CSN). (***B***) As a positive control, we observe Cbln1 protein in the parafascicular nucleus of the thalamus, consistent with prior reports using the Cbln1 E3 antibody (Wei et al., 2007; Kusnoor et al., 2010). In confocal insets (A*, ***A^#^***, B*), yellow arrowheads indicate examples of Cbln1+, Ctip2+ cells (CSN_TL_), blue arrowheads indicate examples of Cbln1+, Ctip2− cells, and pink arrowheads indicate examples of Cbln1−, Ctip2+ cells (CSN_BC-med_). Apparent Cbln1 antibody staining in the upper layers of the somatosensory cortex is not specific when compared with secondary antibody only negative control staining. Sections were also stained for DAPI (blue, nuclei). Scale bars are 500 µm.

To determine whether Cbln1 is translated in CSN_TL_, we performed immunocytochemistry for Cbln1 and Ctip2 on coronal WT brain sections at P4 (Fig. 5). As a positive control, we observe high levels of Cbln1 protein in the parafascicular nucleus (PF) of the thalamus (Fig. 5*B*) as previously reported (Kusnoor et al., 2010); this is consistent with high *Cbln1* expression levels in the PF by fluorescence *in situ* hybridization (Fig. 4*D*). In the neocortex, Cbln1 protein is found in both the cingulate cortex and in medial Layer V, and excluded from lateral Layer V as expected (Fig. 5*A*). We find that a majority of Cbln1-expressing neurons in medial Layer V are high Ctip2+ (Fig. 5*A*). Intriguingly, unlike in the PF where Cbln1 is found in the cytoplasm (Fig. 5*B*), Cbln1 appears relatively localized within the nucleus in CSN (Fig. 5*A*), which has not been previously reported (see Discussion). Taken together, the localization, temporal trajectory, and cell type identity of Cbln1 expression and protein levels suggest that Cbln1 might function during CSN_TL_ axon extension.

### Other cerebellin family members are not expressed by CSN

Cbln1 forms homo- and hetero-complexes with itself or with other members of the Cbln family, and these complexes are known to perform varied, compensatory, and redundant functions (Pang et al., 2000a; Bao et al., 2006; Iijima et al., 2007; Miura et al., 2009; Joo et al., 2011; Rong et al., 2012; Seigneur and Südhof, 2018; Seigneur et al., 2018). To determine whether other *Cbln* family members are expressed by CSN_medial_ and might potentially interact with *Cbln1* in CSN_medial_, we examined previously published gene expression datasets and performed *in situ* hybridization in wild-type mice.

**Figure 6.**
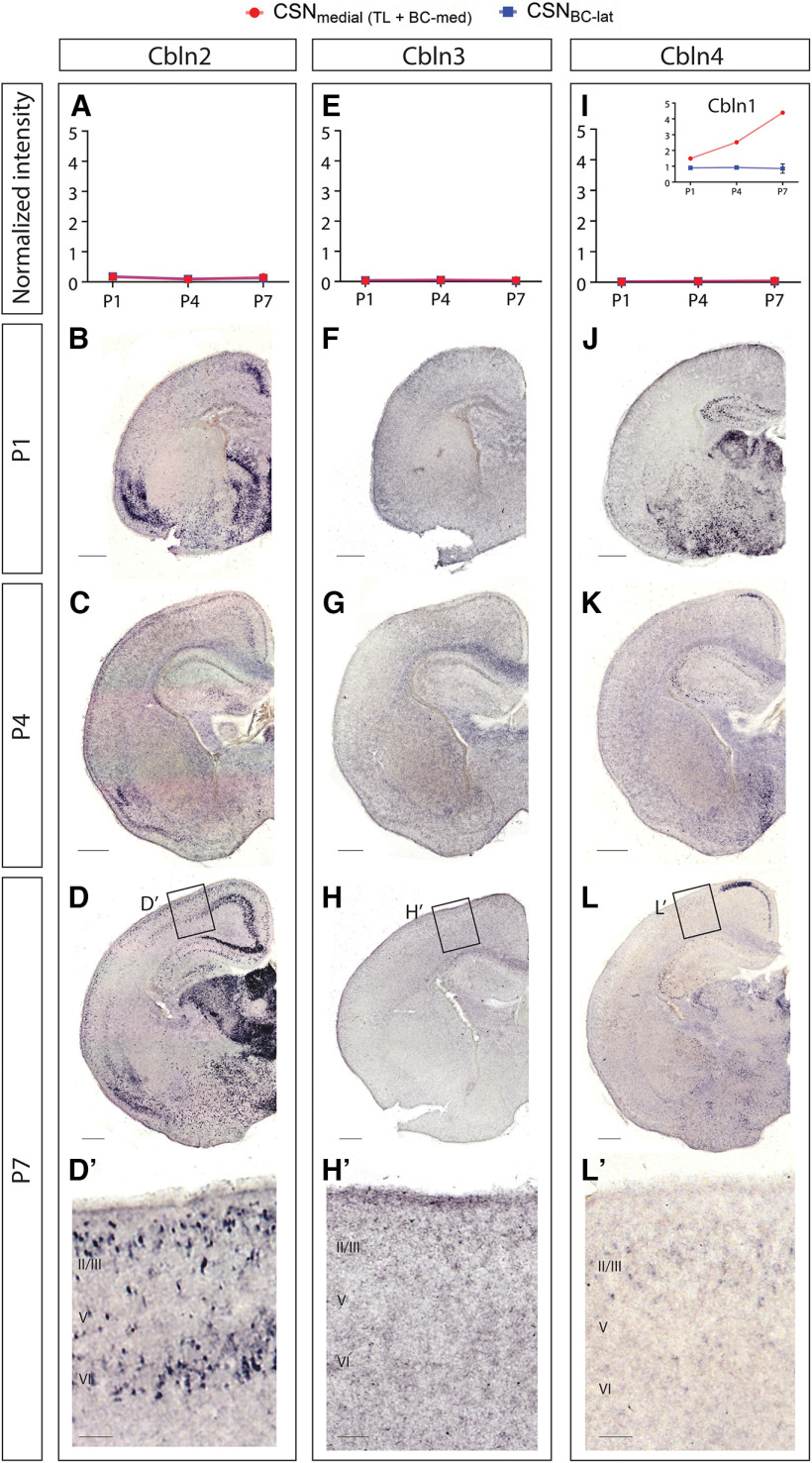
Other *Cbln* family members are not expressed by developing CSN. Prior differential gene expression analysis (Sahni et al., 2021a) identified *Cbln1* (inset in ***I***) as expressed by CSN_medial_. In that dataset, *Cbln2* (***A***), *Cbln3* (***E***), and *Cbln4* (***I***) exhibit no expression by CSN_medial_ (blue) or CSN_BC-lat_ (red) at P1, P4, and P7. Chromogenic *in situ* hybridization at P1 (***B***, ***F***, ***J***), P4 (***C***, ***G***, ***K***), and P7 (***D***, ***H***, ***L***) confirms that *Cbln3* and *Cbln4* are not expressed in Layer V of the developing neocortex and that *Cbln2* is mostly excluded from Layer V. Neurons expressing *Cbln2* in Layer V are likely callosal projection neurons (Arlotta et al., 2005). Scale bars are 100 µm for insets and 500 µm for other images.

Differential gene expression analysis comparing CSN_BC-lat_ and CSN_medial_ indicates that *Cbln2*, *Cbln3*, and *Cbln4* are not expressed, or expressed at very low levels, by either CSN_BC-lat_ or CSN_medial_ during early postnatal development (Sahni et al., 2021a; Fig. 6*A*,*E*,*I*). Accordingly, there is also no detectable difference between *Cbln2*, *Cbln3*, or *Cbln4* expression in CSN_BC-lat_ compared with CSN_medial_, suggesting that these genes are unlikely to function in a CSN subpopulation-specific manner. Interestingly, prior gene expression comparisons at late embryonic and early postnatal times found that *Cbln2* expression is enriched in a different neocortical projection neuron subtype, callosal projection neurons (CPNs), as compared with CSN, and that *Cbln3* and *Cbln4* are not expressed by CPN or by CSN (Arlotta et al., 2005). Together, these datasets suggest that Cbln1 functions independently of other Cbln family members within CSN_medial_.

Chromogenic *in situ* hybridization in wild-type mice confirmed the differential gene expression analyses. As reported previously (Miura et al., 2006; Seigneur and Südhof, 2017), *Cbln2* is expressed at high levels in cingulate and piriform cortex, and at intermediate levels in Layer VI at P1, P4, and P7 (Fig. 6*B–D*). A small fraction of Layer V neurons express *Cbln2* at P1 (Fig. 6*B*), but these are likely CPN based on the previous transcriptomic analysis (Arlotta et al., 2005). *Cbln3* is not expressed within sensorimotor cortex at P1, P4, or P7 (Fig. 6*F–H*). Similarly, *Cbln4* is not expressed in Layer V, but is expressed in the cingulate at P4 and P7 (Fig. 6*J–L*). Together with the transcriptomic analyses, these results indicate that *Cbln2*, *Cbln3*, and *Cbln4* expression is likely absent in CSN, and thus, suggest that Cbln1 function in CSN_medial_ during early postnatal development is independent of other Cbln family members.

### Cbln1 is not required for CSN_TL_ axon extension to the thoraco-lumbar spinal cord

**Figure 7.**
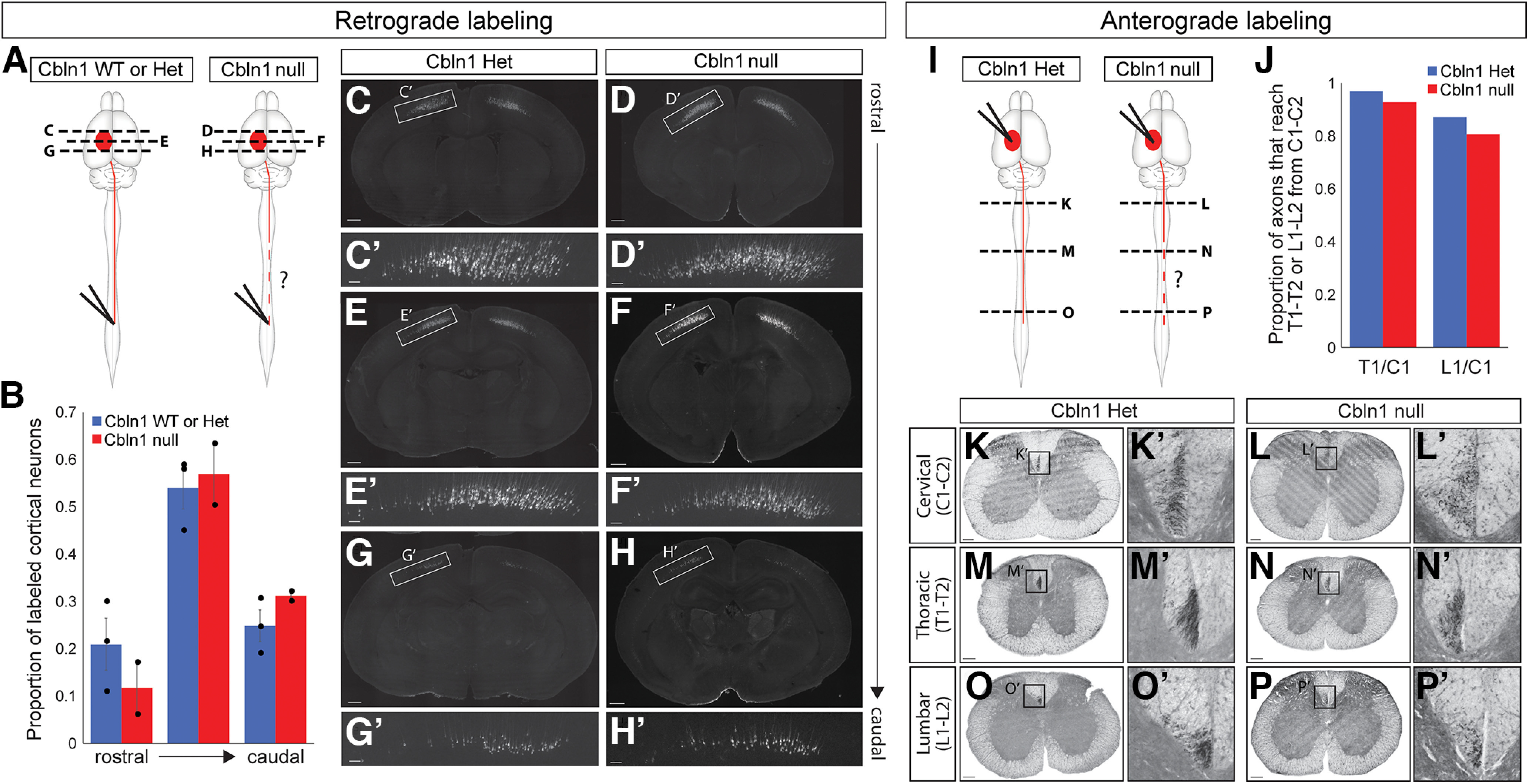
Cbln1 is not required for axon extension to lumbar L1–L2. ***A***, Experimental outline: Retrograde labeling with injection of CTB-555 at lumbar L1–L2 was performed in *Cbln1* WT, *Cbln1* Het, and *Cbln1* null mice at P5, and the number of retrogradely labeled neurons throughout the rostro-caudal extent of medial sensorimotor cortex was analyzed at P7. ***B***, There is no qualitative difference in the proportion of retrogradely labeled CSN in medial sensorimotor cortex at rostral, middle, or caudal levels between *Cbln1* WT or Het (n=3) and *Cbln1* null (n=2) mice. ***C–H***, Representative rostral, middle, and caudal sections from a *Cbln1* Het and a *Cbln1* null mouse. Scale bars are 100 µm for insets and are 500 µm for all other images. ***I***, Experimental outline. Anterograde labeling via biotinylated dextran amine (BDA) iontophoresis into caudomedial sensorimotor cortex was performed in *Cbln1* Het and *Cbln1* null mice at P28. The number of BDA-labeled axons at cervical C1–C2, thoracic T1–T2, and lumbar L1–L2 was counted at P35 for three axial sections per spinal level, and the average number of counts is plotted. ***J***, There is no qualitative difference in the proportion of axons that reach thoracic T1–T2 or lumbar L1–L2 from cervical C1–C2 between *Cbln1* Het and *Cbln1* null mice. ***K–P***, Representative axial sections from cervical C1–C2, thoracic T1–T2, and lumbar L1–L2 in *Cbln1* Het and *Cbln1* null mice. Scale bars are 100 µm.

Since *Cbln1* expression levels coincide with the peak period of CSN_TL_ axon extension to thoracic and lumbar segments, we next investigated whether Cbln1 is necessary for CSN_TL_ axon extension to these distal spinal segments. We analyzed *Cbln1* null mice using anterograde and retrograde labeling (Hirai et al., 2005). We injected the retrograde tracer cholera toxin subunit B conjugated with Alexa-Fluor 555 (CTB-555) into lumbar L1–L2 in *Cbln1* wild-type (WT), *Cbln1* heterozygous (Het), and *Cbln1* null mice at P5, at which time the first CSN_TL_ axons have reached the lumbar cord, and analyzed the number of retrogradely labeled CSN in sensorimotor cortex at P7 (Fig. 7*A*). As expected, labeled CSN are located caudomedially in sensorimotor cortex. We observe no qualitative difference in the overall distribution or number of retrogradely labeled CSN in *Cbln1* null mice (Fig. 7*B–H*).

Next, we injected the anterograde tracer biotinylated dextran amine (BDA) into the caudomedial sensorimotor cortex at P28 and analyzed the brain and spinal cord at P35 to determine whether *Cbln1* might be required for the maintenance of lumbar axon extension (Fig. 7*I*). Once again, there is no qualitative difference in the proportion of CSN axons at cervical C1–C2 that reach thoracic T1–T2 or lumbar L1–L2 between *Cbln1* WT and *Cbln1* null mice (Fig. 7*J–P*). These results suggest that *Cbln1* is not necessary for CSN_TL_ axon extension to the thoracic and lumbar cord.

### Misexpression of Cbln1 in CSN_BC-lat_ leads to aberrant axon extension past the cervical cord toward distal thoraco-lumbar spinal segments

Although Cbln1 is not required for CSN_TL_ axon extension to distal thoraco-lumbar spinal segments, we wondered whether Cbln1 might be sufficient to direct long CSN axon extension. We had previously established that misexpression of Crim1, a CSN_TL_-specific control, in CSN_BC-lat_ can redirect a subset of their axons to caudal thoracic segments (Sahni et al., 2021b). We therefore investigated whether Cbln1 is similarly sufficient to direct thoraco-lumbar axon extension by CSN_BC-lat_, which normally do not express *Cbln1*. To test this hypothesis, we introduced a plasmid expressing *Cbln1* and an EGFP reporter into CSN_BC-lat_ via *in utero* electroporation at E12.5. Control mice received a plasmid expressing EGFP alone (schematized in Fig. 6*A*). CSN_BC-lat_ axons normally reach the caudal cervical cord by P1 and never extend past the rostral-most segments in the thoracic cord (Sahni et al., 2021a). We examined electroporated mice at P4, by which time the most distally extending CSN_BC-lat_ axons have normally terminated within the caudal cervical or rostral-most segments of the thoracic cord.

**Figure 8.**
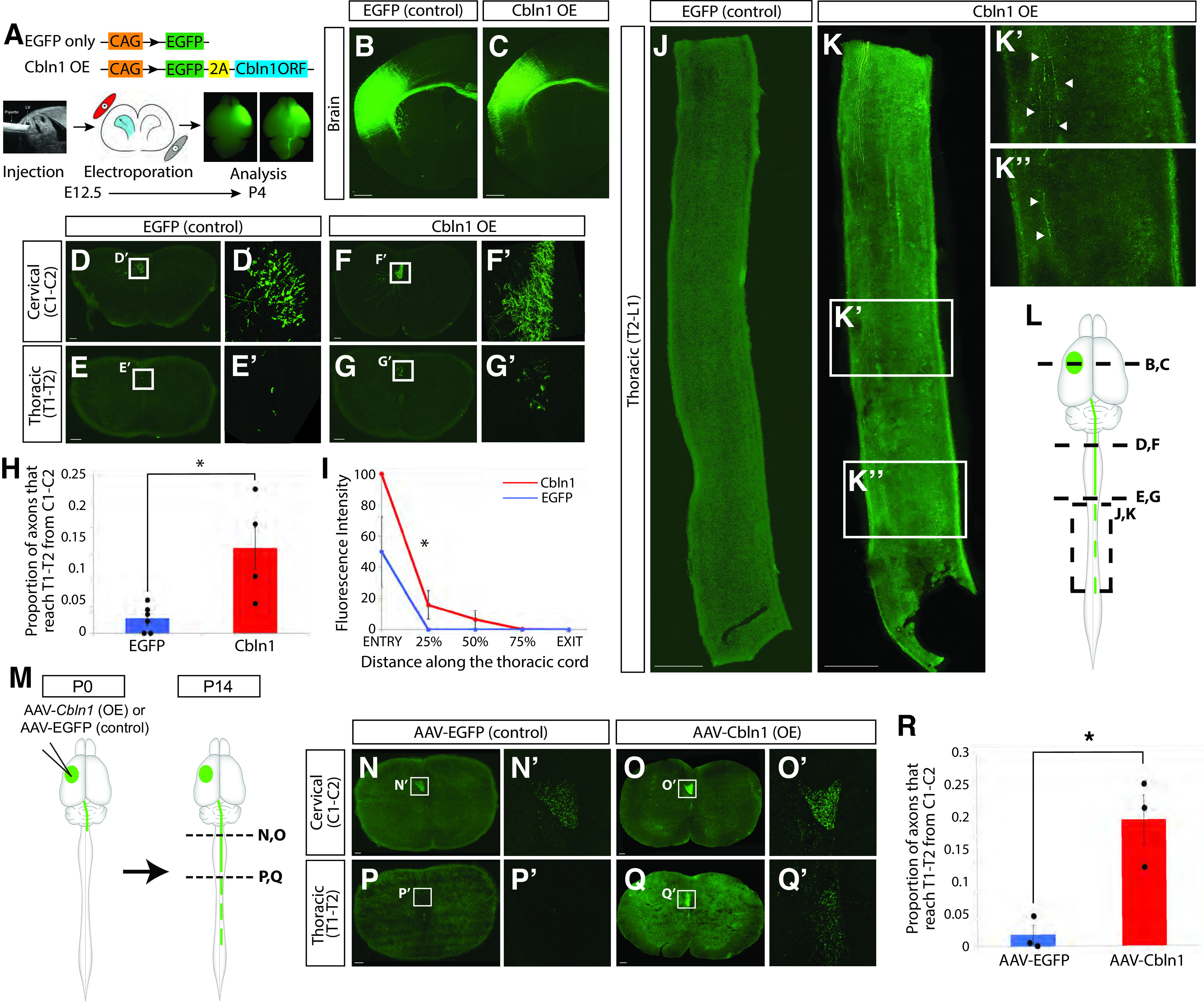
Cbln1 overexpression in CSN_BC-lat_ is sufficient to redirect axon extension to distal thoracic spinal segments. ***A***, Experimental outline: In one set of experiments, plasmids were designed to express either *EGFP* alone, or both *EGFP* and *Cbln1* (Cbln1 OE). The constructs were delivered to developing CSN_BC-lat_ in lateral cortex using *in utero* electroporation at E12.5, and tissue was collected at P4 for analysis. CSN_BC-lat_ axons were visualized using immunocytochemistry for EGFP. ***B***, ***C***, Electroporation location and distribution in the brain are well-matched between EGFP (control) and Cbln1 OE mice. ***D–G***, The number of CSN_BC-lat_ axons at cervical C1–C2 and thoracic T1–T2 were counted in axial sections of the spinal cord in EGFP and Cbln1 OE mice. ***H***, The proportion of individual axons that reach thoracic T1–T2 from cervical C1–C2 is significantly higher in Cbln1 OE (red, n=4) compared with EGFP (control; blue, n=6) mice (p=0.01 by two-tailed Student's *t* test). ***I***, CST fluorescence intensity was quantified along the rostro-caudal extent of the thoracic cord and normalized to the fluorescence intensity at thoracic T2. Significantly more *Cbln1*-expressing CSN_BC-lat_ axons extend into the distal thoracic cord when compared with EGFP controls (p=0.04 by two-way ANOVA with repeated measures followed by Fisher's least significant difference *post hoc* test). ***J***, ***K***, CSN_BC-lat_ axons extend into the distal thoracic cord in thoracic sagittal sections in Cbln1 OE (arrowheads in ***K'***, ***K”***) but not in EGFP (control) mice. ***L***, Schematic indicating anatomic location of sections displayed in ***B–K***. ***M***, Experimental outline: In a second set of experiments, AAV particles engineered to express either *EGFP* alone (AAV-EGFP) or both *EGFP* and *Cbln1* (AAV-Cbln1) were injected into rostrolateral sensorimotor cortex at P0 to test whether *Cbln1* is sufficient to redirect axon extension by postmitotic CSN_BC-lat_. AAV-injected mice were then analyzed at P14. ***N–Q***, The number of axons that reach cervical C1–C2 and thoracic T1–T2 were counted in axial sections of the spinal cord in AAV-EGFP and AAV-Cbln1 mice. ***R***, There are significantly more axons that reach thoracic T1–T2 from cervical C1–C2 in AAV-Cbln1 (red, n=3) compared with AAV-EGFP (blue, n=3; p=0.02 by one-tailed Student's *t* test). Scale bars are 100 µm for ***D–G*** and ***N–Q*** and 500 µm for ***B***, ***C***, ***J***, and ***K***. Each data point in ***H*** and ***R*** is the axon count averaged over three axial sections per spinal level per mouse.

We first confirmed that all electroporations were restricted to lateral sensorimotor cortex where CSN_BC-lat_ reside (Fig. 8*B*,*C*). We next investigated the percentage of CSN_BC-lat_ axons that reach the rostral thoracic cord (T1–T2) from the rostral cervical cord (C1–C2). Strikingly, when Cbln1 is misexpressed in CSN_BC-lat_, a sixfold higher percentage of axons reach T1–T2 from C1–C2 (T1/C1) compared with the control (16.1%±4.0% for Cbln1 misexpression, 2.8%±1.3% for the control; p=0.01; Fig. 8*D–H*). This indicates that Cbln1 misexpression aberrantly directs a higher percentage of CSN_BC-lat_ axons to extend past their normal cervical targets into the thoracic cord by P4.

We next investigated whether these redirected axons from *Cbln1*-expressing CSN_BC-lat_ extend further caudally toward more distal thoracic segments. As expected, in control mice, CSN_BC-lat_ axons do not extend past the rostral-most segments of the thoracic cord (Fig. 8*J*). In striking contrast, not only do a significantly larger number of CSN_BC-lat_ axons reach the rostral thoracic cord upon Cbln1 misexpression, a subset of these axons reach the distal-most segments of the thoracic cord (T13) at P4 (Fig. 8*K*). Quantification of CST fluorescence intensity along the rostro-caudal extent of the thoracic cord (normalized to the fluorescence intensity at thoracic T2), reveals that significantly more Cbln1-expressing CSN_BC-lat_ axons extend into the distal thoracic cord when compared with controls (p=0.04; Fig. 8*I*). Indeed, no axons even reached T2 in 3 of the 6 control samples (Fig. 8*I*). Interestingly, although axon collateralization by CSN_BC-lat_ is well underway by P4 (Bareyre et al., 2005), we do not observe any collateralization by the aberrantly extended CSN_BC-lat_ axons on Cbln1 misexpression in the thoracic cord (Fig. 8*K*). Together, these data indicate that Cbln1 is sufficient to redirect CSN_BC-lat_ axon extension past the cervical cord toward thoracic spinal segments but does not promote axon collateralization.

### Postnatal misexpression of Cbln1 in CSN_BC-lat_ leads to aberrant long CSN_BC-lat_ axon extension

The time course of *Cbln1* expression suggests that its function is required specifically during the period of CSN_TL_ axon extension. However, since misexpression by *in utero* electroporation begins in progenitors and continues into postmitotic neurons, there remained the unlikely possibility that the effect of Cbln1 misexpression on CSN_BC-lat_ axon extension is because of alterations in early CSN_BC-lat_ specification before their axons reach the spinal cord, ultimately causing their aberrant axon extension later at P4. To directly investigate this possibility, we performed misexpression in CSN_BC-lat_ at P0 via AAV-mediated gene delivery. We generated AAV particles engineered to express Cbln1 and an EGFP reporter (AAV-Cbln1). Control mice received AAV particles engineered to express EGFP alone (AAV-EGFP). We injected these particles into the rostrolateral cortex of P0 mice under ultrasound guided backscatter microscopy and examined CSN_BC-lat_ axon extension at P14 (schematized in Fig. 8*M*), at which point CSN axonal projections have been pruned and the adult connectivity pattern of the CST is largely established (Bareyre et al., 2005; Sahni et al., 2020, 2021a).

As with embryonic misexpression of Cbln1 in CSN_BC-lat_, postnatal Cbln1 misexpression leads to aberrant CSN_BC-lat_ axon extension past the cervical cord into the thoracic spinal cord. We quantified the percentage of axons that reach thoracic T1–T2 from cervical C1–C2 in axial sections from mice injected with either AAV-EGFP or AAV-Cbln1 (Fig. 8*N–R*). There is a significant increase in the percentage of axons that reach thoracic T1–T2 at P14 compared with controls (22.8%±4.5% for Cbln1 misexpression, 2.0%±1.7% for the control; p=0.02; Fig. 8*R*). As with the *in utero* electroporation experiments, these aberrantly extended CSN_BC-lat_ axons on Cbln1 misexpression at P0 also fail to collateralize in the thoracic cord (data not shown). Therefore, postnatal misexpression of Cbln1 at P0 is sufficient to direct CSN_BC-lat_ axons past the cervical cord and into the thoracic cord. Together, these data combined with the results from *in utero* misexpression experiments indicate that Cbln1 regulates axon extension without affecting CSN axon collateralization. This is in contrast to known functions of Cbln1 as a critical synaptic organizer in the cerebellum, striatum, and other brain regions (Hirai et al., 2005; Kusnoor et al., 2010; Seigneur and Südhof, 2018), and represents a unique function of Cbln1 in controlling CSN axon extension independent of effects on axon collateralization or synapse formation.

### Postnatal overexpression of Cbln1 in CSN_medial_ is sufficient to drive long CSN axon extension to the thoraco-lumbar spinal cord

The experiments above indicate that CSN_TL_ axon extension to caudal thoracic and lumbar segments does not require Cbln1 function, but that Cbln1 is sufficient to redirect CSN_BC-lat_ axons to the caudal thoracic cord. Unlike CSN in lateral sensorimotor cortex, which only project to bulbar-cervical segments (CSN_BC-lat_), CSN_medial_ include distinct subpopulations with projections to bulbar-cervical (CSN_BC-med_) or thoraco-lumbar segments (CSN_TL_). Previous work has established that CSN_BC-lat_, CSN_BC-med_, and CSN_TL_ are distinct, molecularly delineated subpopulations that can be distinguished before CSN axons have even reached the spinal cord (schematized in Fig. 1*A*; Sahni et al., 2021a).

*Cbln1* is specifically expressed by CSN_TL_ and not by CSN_BC-med_ within CSN_medial_ (Fig. 4). Since Cbln1 misexpression in CSN_BC-lat_ promotes long axon extension, we next investigated whether Cbln1 is also sufficient to redirect axon extension by CSN_BC-med_, a molecularly and spatially distinct subpopulation from CSN_BC-lat_. Since CSN_BC-med_ and CSN_TL_ reside in a spatially interdigitated manner in caudomedial sensorimotor cortex (Sahni et al., 2021a), we overexpressed Cbln1 in medial cortex. We reasoned that if Cbln1 misexpression redirected CSN_BC-med_ axons past the cervical cord toward caudal thoracic and lumbar segments, we would observe an increase in the proportion of axons that extend to thoracic T1–T2 from cervical C1–C2, similar to our observations with CSN_BC-lat_. However, if Cbln1 overexpression only had an effect in CSN_TL_, we might observe an increase in axon extension to lumbar L1–L2 but not an increase in axon extension to thoracic T1–T2 from cervical C1–C2.

**Figure 9.**
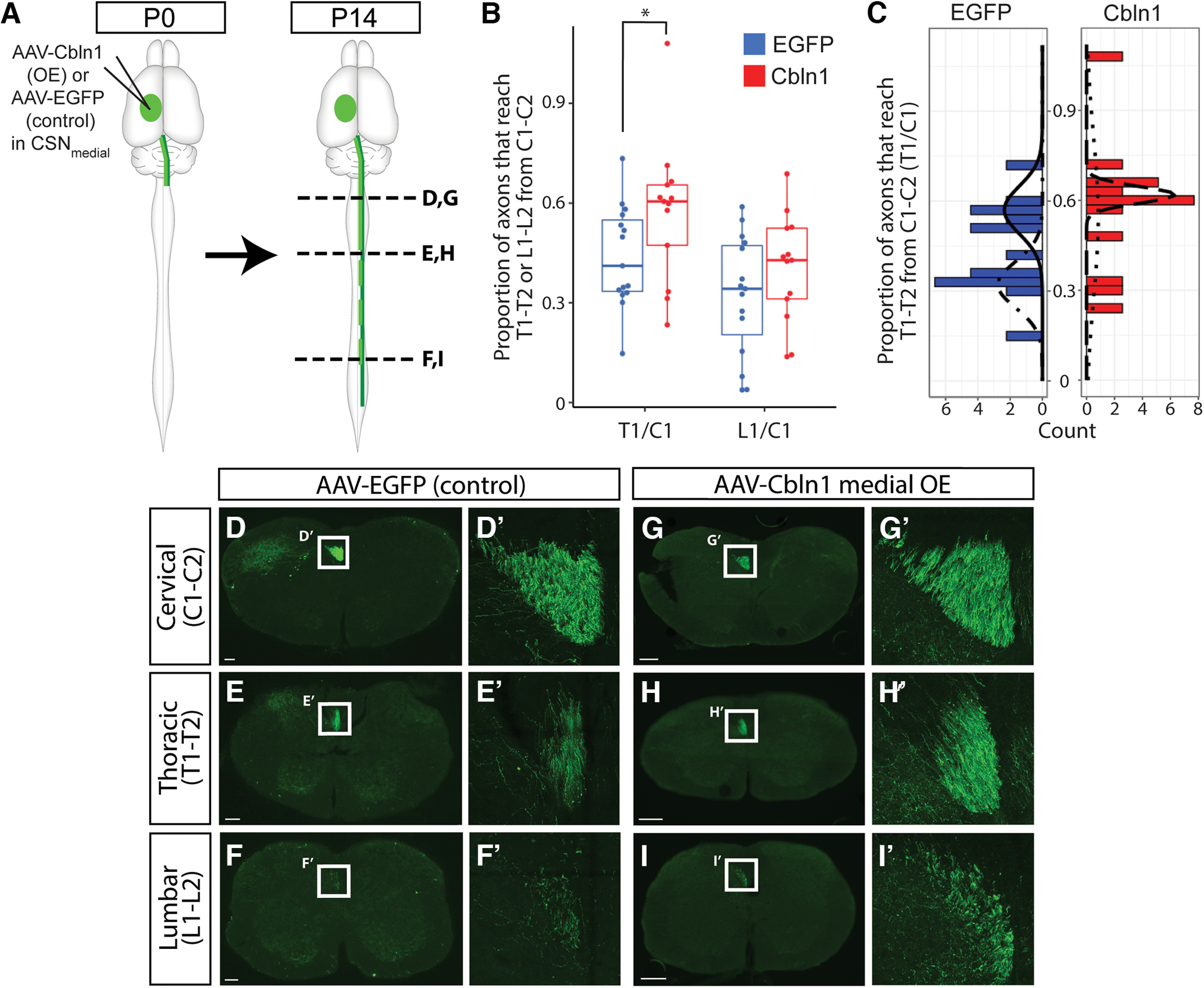
Cbln1 overexpression in CSN_medial_ is sufficient to increase the number of axons extending past the cervical spinal cord. ***A***, Experimental outline: AAV particles engineered to express either *EGFP* alone (AAV-EGFP, control) or both *EGFP* and *Cbln1* (AAV-Cbln1) were injected into medial sensorimotor cortex at P0. AAV-injected mice were analyzed at P14. ***B***, CST intensity was quantified in three axial sections per spinal level (cervical C1–C2, thoracic T1–T2, and lumbar L1–L2) per mouse, and the mean value across three axial sections is plotted. The proportion of axons that reach thoracic T1–T2 from cervical C1–C2 (T1/C1) is significantly higher in AAV-Cbln1 (red, n=13) compared with AAV-EGFP (blue, n=15) (p=0.03 by one-tailed Student's *t* test). In contrast, the proportion of axons that reach lumbar L1–L2 from cervical C1–C2 is not significantly different between AAV-Cbln1 and AAV-EGFP mice (p=0.11 by one-tailed Student's *t* test). ***C***, We modeled the distribution of T1/C1 in control AAV-EGFP or AAV-Cbln1-injected mice as a mixture of two Gaussians. The distribution of T1/C1 in control AAV-EGFP-injected mice appears bimodal with one Gaussian centered at 0.32±0.08 and the other at 0.57±0.08, likely reflecting variability in the proportion of CSN_BC-med_ or CSN_TL_ labeled by each injection. In contrast, the distribution of T1/C1 in AAV-Cbln1-injected mice appears unimodal with one Gaussian centered at 0.54±0.27 and the other centered at 0.62±0.03. These Gaussians are similar to the Gaussian with control AAV-EGFP injections centered at 0.57, which is likely comprised of a higher proportion of CSN_TL_ compared with CSN_BC-med_. This suggests that Cbln1 overexpression might specifically shift the segmental targeting of CSN_BC-med_ past the bulbar-cervical cord into the thoraco-lumbar cord, but not overtly affect the segmental targeting of CSN_TL_. ***D–I***, Representative axial sections from cervical C1–C2, thoracic T1–T2, and lumbar L1–L2 from control AAV-EGFP and AAV-Cbln1-injected mice. Scale bars are 100 µm.

We overexpressed Cbln1 at P0 via AAV-mediated gene delivery in medial sensorimotor cortex, where *Cbln1* is normally expressed by only CSN_TL_. We injected the medial cortex of P0 mice with either AAV particles engineered to express Cbln1 and an EGFP reporter (AAV-Cbln1) or AAV particles engineered to express EGFP alone (AAV-EGFP). We examined CSN_medial_ axon extension at P14 by quantifying CST fluorescence intensity at thoracic T1–T2 and lumbar L1–L2 as a proportion of the fluorescence intensity at cervical C1–C2 in axial sections from mice injected with either AAV-Cbln1 or AAV-EGFP (schematized in Fig. 9*A*).

Since there is no positive molecular identifier for CSN_BC-med_, we investigated their axon targeting as a subset of the broader CSN_medial_ subpopulation. Given the two-population diversity of CSN_medial_, injections in medial sensorimotor cortex will label varying proportions of CSN_BC-med_ and CSN_TL_, since both subpopulations reside interdigitated in medial sensorimotor cortex. Therefore, we expected these injections to result in greater variability in the proportion of labeled CSN_medial_ axons that reach thoracic T1–T2 from cervical C1–C2 (we refer to this proportion as T1/C1) and, as a result, analyzed a larger number of mice to account for this variability (n=13 for AAV-Cbln1 injections; n=15 for control AAV-EGFP injections). Indeed, we observe high variance in the distribution of T1/C1 following injection with control AAV-EGFP (Fig. 9*B*). Importantly, the AAV-EGFP-injected mice clustered broadly into two groups, likely reflecting the predicted differences in the extent of labeling between CSN_BC-med_ and CSN_TL_ in each individual mouse. Therefore, we modeled the distribution of T1/C1 as a mixture of two Gaussians (Fig. 9*C*). The distribution of T1/C1 following the injection of control AAV-EGFP appears bimodal, with one Gaussian centered at 0.32±0.08, likely reflecting a higher proportion of CSN_BC-med_ relative to CSN_TL_ in these injections, and the other Gaussian at 0.57±0.08, likely reflecting a higher proportion of CSN_TL_ relative to CSN_BC-med_.

Despite these differences in the relative numbers of CSN_BC-med_ and CSN_TL_ labeled by each injection, we investigated whether Cbln1 overexpression is sufficient to alter the T1/C1 proportion in AAV-Cbln1-injected mice. As with misexpression of Cbln1 in CSN_BC-lat_, we find that overexpression of Cbln1 in CSN_medial_ leads to long CSN axon extension past the cervical cord to thoraco-lumbar spinal segments (Fig. 9*B–I*). There is a significant increase in T1/C1 (57.5%±5.9% for Cbln1 overexpression, 43.8%±3.9% for the control; p=0.03). Intriguingly, we do not find a similar increase in the proportion of axons that reach lumbar L1–L2 from cervical C1–C2 (40.3%±4.5% for Cbln1 overexpression, 32.1%±4.7% for the control; p=0.11) on Cbln1 overexpression in CSN_medial_.

In contrast with the T1/C1 distribution in control AAV-EGFP-injected mice, which appears bimodal, the distribution of T1/C1 following Cbln1 overexpression appears unimodal with the center of both Gaussian distributions around 0.57 (Fig. 9*C*). This is similar to the Gaussian distribution in the control T1/C1 distribution that is likely enriched for CSN_TL_. Although this analysis does not entail *a priori* cell identification, it suggests that Cbln1 overexpression might specifically shift the segmental targeting of CSN_BC-med_ past the bulbar-cervical cord into the thoraco-lumbar cord, but might not substantially affect the segmental targeting of CSN_TL_. Together with our findings that Cbln1 misexpression in CSN_BC-lat_ promotes axon extension past their normal cervical targets, these results indicate that Cbln1 is sufficient to drive axon extension past the cervical cord by multiple spatially and molecularly distinct CSN subpopulations.

### Cbln1 is regulated by Klhl14 but acts independently of Crim1 to control CSN long axon extension

We previously identified Klhl14 and Crim1 as molecular controls expressed by CSN_BC-lat_ and CSN_TL_, respectively (Sahni et al., 2021a, b). Klhl14 functions to limit CSN_BC-lat_ axons to proximal segments in the cervical spinal cord during early postnatal development. Knock-down of *Klhl14* by shRNA leads to aberrant CSN_BC-lat_ axon extension toward distal thoracic segments at P4. This aberrant axon extension is accompanied by an upregulation of *Crim1* by CSN_BC-lat_.

**Figure 10.**
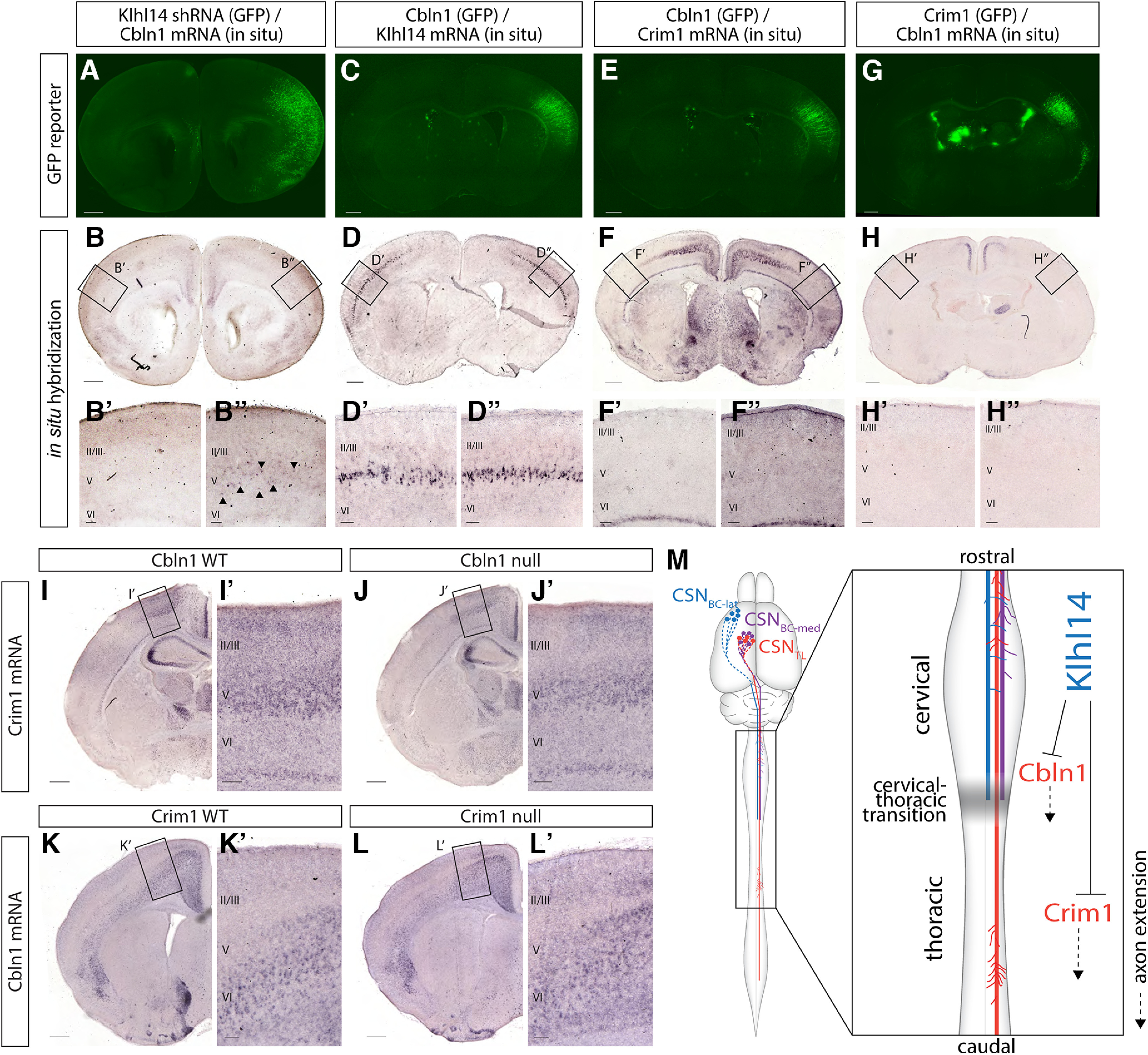
Cbln1 expression is regulated by Klhl14 but not by Crim1. ***A***, ***B***, Coronal section of a P4 brain that was electroporated *in utero* at E12.5 with Klhl14 shRNA. ***A***, EGFP fluorescence (green) shows the site of electroporation in lateral cortex. ***B***, *In situ* hybridization image of the same section in (***A***) shows *Cbln1* expression. *Cbln1* is normally restricted to medial cortex. However, *Klhl14* knock-down by shRNA causes ectopic *Cbln1* expression in lateral cortex (arrowheads in ***B”***) in the electroporated cortical hemisphere (compare ***B”*** with contralateral ***B'***). ***C***, ***D***, Coronal section of a P4 brain that was electroporated *in utero* at E12.5 with a plasmid containing *Cbln1* and *EGFP* (Cbln1-EGFP). ***C***, EGFP fluorescence (green) shows the site of electroporation in lateral cortex. ***D***, *In situ* hybridization image of the same section in ***C*** showing *Klhl14* expression in Cbln1-misexpressing CSN_BC-lat_ remains unchanged, indicating that *Klhl14* expression in lateral Layer V is unaffected by Cbln1 misexpression. ***E***, ***F***, Coronal section of a P4 brain that was electroporated *in utero* at E12.5 with Cbln1-EGFP. ***E***, EGFP fluorescence (green) shows the site of electroporation in lateral cortex. ***F***, *In situ* hybridization image of the same section in ***E*** showing that there is no ectopic *Crim1* expression in Cbln1-misexpressing CSN_BC-lat_. ***G***, ***H***, Coronal section of a P4 brain that was electroporated *in utero* at E12.5 with Crim1-EGFP. *Crim1* misexpression in CSN_BC-lat_ can redirect axons toward caudal thoracic spinal segments (Sahni et al., 2021b). ***G***, EGFP fluorescence (green) shows the site of electroporation in lateral cortex. ***H***, *In situ* hybridization image of the same section in (***G***) showing that there is no ectopic *Cbln1* expression in Crim1-misexpressing CSN_BC-lat_. ***I***, ***J***, *Crim1* expression in medial Layer V does not differ between *Cbln1* WT and *Cbln1* null mice. ***K***, ***L***, *Cbln1* expression in medial Layer V does not differ between *Crim1* WT and *Crim1* null mice. ***M***, Summary schematic displaying molecular controls over CSN axon extension both at, and beyond, the transition between cervical and thoracic spinal segments. Together with the previous investigations identifying Crim1 and Klhl14 function (Sahni et al., 2021b), our data suggest a model whereby Cbln1 directs CSN axon extension from the cervical into the thoracic cord, whereas Crim1 directs those CSN axons that cross this transition zone to extend further toward caudal thoracic and lumbar spinal segments. This indicates that CSN segmental axon targeting toward thoracic and lumbar segments involves multiple, distinct molecular regulators acting at distinct spinal levels. *Klhl14*, which is specifically expressed in CSN_BC-lat_ and restricts CSN_BC-lat_ axon extension to the bulbar-cervical segments, represses the expression of both *Cbln1* and *Crim1* in CSN_BC-lat_. This indicates that Klhl14 represses a broad program of thoraco-lumbar directed axon extension in CSN_BC-lat_. This program, mediated by multiple independent molecular controls, would otherwise direct CSN axons past the cervical cord toward caudal thoracic and lumbar segments. Scale bars are 100 µm for insets and 500 µm for all other images.

To determine whether Klhl14 might also similarly modulate *Cbln1* expression, we examined coronal brain sections from mice at P4 in which *Klhl14* shRNA was introduced into CSN_BC-lat_ via *in utero* electroporation at E12.5 (Fig. 10*A*). Strikingly, *Cbln1* is ectopically expressed in Layer V in rostrolateral sensorimotor cortex where *Klhl14* is reduced, but not in the contralateral cortex where *Klhl14* expression is normal (Fig. 10*B*). This strongly suggests that *Cbln1* and *Crim1* expression are both repressed by Klhl14 in CSN_BC-lat_ to regulate CSN axon extension.

We also examined whether Cbln1 overexpression can regulate *Klhl14* expression. Coronal brain sections from mice electroporated with *Cbln1* in lateral cortex at E12.5 and analyzed at P4 were examined for *Klhl14* expression via *in situ* hybridization (Fig. 10*C*). There is no difference in *Klhl14* expression in the electroporated versus contralateral cortex (Fig. 10*D*). This strongly suggests that Klhl14 acts upstream of *Cbln1* to repress *Cbln1* expression by CSN_BC-lat_.

Gain-of-function experiments show that Crim1 and Cbln1 function at distinct levels of the spinal cord. Crim1 misexpression does not increase the proportion of CSN_BC-lat_ axons that reach thoracic T1–T2, but it does redirect the small minority of CSN_BC-lat_ axons that reach the thoracic cord to extend farther into the thoracic cord toward caudal thoracic segments (Sahni et al., 2021b). In contrast, Cbln1 misexpression significantly increases axon extension to thoracic T1–T2 by both CSN_BC-lat_ and CSN_medial_. These data suggest that Cbln1 and Crim1 function in distinct pathways, and that Klhl14 acts as an upstream regulator of both *Cbln1* and *Crim1*.

These results led us to investigate whether *Cbln1* and *Crim1* control CSN long axon extension via the same or distinct genetic pathways. We first examined *Crim1* expression in sensorimotor cortex in *Cbln1* WT and *Cbln1* null mice. We detect no differences in *Crim1* expression (Fig. 10*I*,*J*). Similarly, we detect no difference in *Cbln1* expression in sensorimotor cortex between *Crim1* WT and *Crim1* null mice (Fig. 10*K*,*L*). We further investigated whether Cbln1 misexpression in CSN_BC-lat_ via *in utero* electroporation at E12.5 might modulate *Crim1* expression when analyzed at P4, and vice versa. There is no difference in *Crim1* expression between *Cbln1*-expressing CSN_BC-lat_ compared with the contralateral cortex (Fig. 10*E*,*F*). There is also no difference in *Cbln1* expression between *Crim1*-expressing CSN_BC-lat_ and the contralateral cortex (Fig. 10*G*,*H*). Together, these data indicate that *Crim1* and *Cbln1* act via distinct genetic pathways to control CSN axon extension.

## Discussion

Previous work has identified that CSN subpopulations exhibit striking axon extension specificity during development, and that this specificity is durably maintained into maturity (Sahni et al., 2021a,b). CSN subpopulations with distinct spinal segmental targets are molecularly distinct from the earliest stages of axon extension, even before their axons reach the spinal cord. We previously investigated two molecular controls, Klhl14 and Crim1, that both prospectively identify CSN subpopulations with segmentally distinct projections, and control these projections. We identified their critical functions in directing CSN axons to appropriate spinal segmental levels, with dual-directional, complementary regulation toward thoraco-lumbar extension (by Crim1) and limiting axon extension past bulbar-cervical segments (by Klhl14). These results indicate that CSN-intrinsic molecular controls, at least in part, govern CSN axonal targeting specificity (Sahni et al., 2021a,b).

Here, we build on this work to identify a novel role for a member of the cerebellin family, Cbln1, in controlling CSN segmental axonal projection targeting. We find that within CSN, *Cbln1* is expressed specifically by CSN_TL_ in medial sensorimotor cortex. The time course of Cbln1 expression by CSN closely aligns with the period of CSN_TL_ axon extension to thoracic and lumbar segments. Misexpression of Cbln1 in CSN_BC-lat_ via either *in utero* electroporation at E12.5 or AAV injection at P0 redirects CSN_BC-lat_ axons past their normal cervical targets to distal segments in the thoraco-lumbar cord. Similarly, Cbln1 overexpression in CSN_medial_ is sufficient to increase the proportion of CSN_medial_ axons that extend to thoracic spinal segments. These results indicate that Cbln1 can direct long axon extension by multiple CSN subpopulations residing in spatially distinct locations in sensorimotor cortex.

This represents a novel function for Cbln1 in axon extension, independent of its well-described function as a synaptic organizer. Cbln1 has been characterized extensively in the cerebellum, where it is localized and secreted at presynaptic terminals of cerebellar granule cells, and is instructive for synapse formation between Purkinje cells and the parallel fibers of granule cells (Hirai et al., 2005; Matsuda et al., 2010; Uemura et al., 2010; Ibata et al., 2019). Outside of the cerebellum, Cbln1 has also been shown to play critical roles in both synapse formation and synapse maintenance in a number of other brain regions, including the hippocampus, striatum, and the ventral tegmental area of the midbrain (Kusnoor et al., 2010; Krishnan et al., 2017; Seigneur and Südhof, 2018).

Strikingly, we find that its function in CSN is quite distinct. *Cbln1* expression by CSN is strongest during the time period of axon extension, before axon collateralization or synapse formation. Moreover, although Cbln1 misexpression in CSN_BC-lat_ redirects these axons to the thoracic cord, these redirected axons do not collateralize in the thoracic cord at either P4 or P14. This suggests that the function and mechanism of Cbln1 in axon extension occurs independent of synapse formation by CSN, raising the possibility that Cbln1 and other classical synaptic organizers might perform currently unappreciated functions in axon extension during development.

Cbln1 is normally thought to be trafficked to presynaptic terminals where it directly affects synapse formation and maintenance in multiple brain regions (Matsuda et al., 2010; Otsuka et al., 2016). It is possible that Cbln1 is similarly trafficked along CSN axons to directly regulate axon extension. Interestingly, our immunocytochemistry results identify Cbln1 protein in the nucleus of CSN_TL_; Cbln1 has not been previously detected in the nucleus in any other contexts. This could potentially suggest a novel nuclear function for Cbln1. For instance, Cbln1 may act as a nuclear scaffold, similar to known functions of C1q family members as scaffolds at the plasma membrane and at the synapse (Innamorati et al., 2002; Elegheert et al., 2016). We note that the Cbln1 E3 antibody used in this study detects the full-length form of Cbln1 (specifically, the globular C1q domain) and not the cleaved cerebellin peptide (Bao et al., 2005). Future investigation into the cellular localization and function of the full-length Cbln1 protein and the smaller cerebellin peptide will likely elucidate: (1) a potentially new role for Cbln1 in the nucleus; (2) whether Cbln1 or its cleaved cerebellin peptide is trafficked along CSN axons; and (3) how Cbln1 or the cerebellin peptide might function to promote long CSN axon extension.

Understanding Cbln1 localization will also be important to guide future work to identify proteins that interact with Cbln1 to mediate its function in regulating CSN axon extension. Cbln1 interactors such as GluRδ2 and β-neurexins have been identified in the cerebellum (Matsuda et al., 2010; Uemura et al., 2010; Cheng et al., 2016; Elegheert et al., 2016), and there is evidence that, in the cerebral cortex, where GluRδ2 is not expressed, Cbln1 instead interacts with GluRδ1 (Matsuda et al., 2010; Rong et al., 2012; Wei et al., 2012; Yasumura et al., 2012). *GRID1* and *GRID2* (which encode GluRδ1 and GluRδ2, respectively), as well as neurexin family members, are expressed in the human spinal cord (GTEx Consortium, 2013). These interactors have not been implicated previously in axon extension; however, it is possible that they might also interact with Cbln1 to promote long axon extension in this novel context.

Although Cbln1 overexpression robustly directs long CSN axon extension, we do not observe any qualitative defects of CSN_TL_ axon extension to thoracic and lumbar spinal segments in *Cbln1* null mice. Given our limited sample size because of complexities related to the COVID-19 pandemic, it is possible that Cbln1 might have more subtle roles over CSN axon extension that might be elucidated by future studies. Nevertheless, our results suggest that Cbln1 is sufficient but not overtly necessary for CSN long axon extension. What might compensate for Cbln1 function in CSN_TL_ in *Cbln1* null mice? The other cerebellin family members, *Cbln2*, *Cbln3*, and *Cbln4*, are known to interact with *Cbln1* and perform compensatory or redundant functions (Pang et al., 2000a; Bao et al., 2006; Iijima et al., 2007; Miura et al., 2009; Joo et al., 2011; Rong et al., 2012; Seigneur and Südhof, 2018; Seigneur et al., 2018). For instance, *Cbln1* and *Cbln2* are normally expressed in the cerebellum. Whereas *Cbln1* null mice are ataxic and have disrupted synaptic connectivity, *Cbln2* null mice display no functional or anatomic deficits in the cerebellum. Interestingly, however, Cbln2 overexpression in the cerebellum of *Cbln1* null mice can rescue their ataxic phenotype, suggesting partial redundancy of function (Rong et al., 2012). Although compensation by other cerebellins presents a tempting hypothesis, we find that *Cbln2*, *Cbln3*, and *Cbln4* are not normally expressed by CSN_medial_ at the time when their axons are normally extending to distal spinal segments. It is possible that molecular controls other than Cbln protein family members might compensate for loss of Cbln1 in CSN_medial_. For instance, Cbln1 and neuroligin-3 have been shown to partially compensate for each other at calyx of Held synapses (Yuzaki, 2017; Zhang et al., 2017). Future studies will be needed to elucidate molecular mechanisms that might compensate for the loss of Cbln1 function in directing CSN axon extension.

We also investigated whether Cbln1 might regulate or be regulated by previously identified molecular controls over CSN axon extension. We previously identified that Klhl14, which is specifically expressed by CSN_BC-lat_ and limits their axon extension to the cervical cord, acts at least in part to repress *Crim1* expression by CSN_BC-lat_. Crim1 misexpression in CSN_BC-lat_ is sufficient to redirect their axons to distal thoracic levels (Sahni et al., 2021b). In the work presented here, we find that Klhl14 also represses *Cbln1* expression by CSN_BC-lat_, suggesting that Klhl14 acts as a broad transcriptional repressor to suppress multiple molecular controls that otherwise would direct CSN axons past the cervical cord. However, we find that *Cbln1* and *Crim1* are not in the same genetic pathway in CSN. In either *Cbln1* null or *Crim1* null mice, there is no change in the expression of the other molecular control. Likewise, when either Cbln1 or Crim1 is misexpressed in CSN_BC-lat_, the other molecular control is not ectopically expressed.

Indeed, Cbln1 and Crim1 perform similar, but distinct, functions in directing long CSN axon extension. Misexpression of Cbln1, but not Crim1 (Sahni et al., 2021b), in CSN_BC-lat_ increases the proportion of axons that reach thoracic T1–T2. Further, although both Cbln1 and Crim1 misexpression in CSN_BC-lat_ is sufficient to redirect those axons that reach T1–T2 to extend further into the thoracic cord, their effects on axon extension within the thoracic cord are distinct. While 100% of mice in which Crim1 was misexpressed in CSN_BC-lat_ had axons that reached at least halfway through the thoracic cord (Sahni et al., 2021b), this was true of only 50% of mice in which Cbln1 was misexpressed in lateral cortex. This suggests that Cbln1 might serve as a regulator of axon targeting at the transition between cervical and thoracic spinal segments, whereas *Crim1* primarily functions to drive axon extension distal to this transition (schematized in Fig. 10*M*).

Additionally, CSN_BC-lat_ axons that aberrantly extend in the thoracic cord upon either *Cbln1* or *Crim1* misexpression fail to collateralize in the thoracic spinal gray matter. It is likely that distinct molecular controls are required to interact with extracellular cues specific to the thoraco-lumbar spinal cord to promote axon collateralization. This is consistent with previous indications that axon extension and collateralization are distinctly regulated processes (Kalil and Dent, 2014; Itoh et al., 2021).

Interestingly and potentially relevant, motor neuron diseases (MNDs) such as amyotrophic lateral sclerosis (ALS) and hereditary spastic paraplegia (HSP) do not affect all CSN equally (Bruijn et al., 2004; Strong and Gordon, 2005; Salinas et al., 2008). In bulbar forms of ALS, e.g., brainstem-projecting CSN are affected, while in HSP, lumbar-projecting CSN preferentially degenerate. Although multiple proteins including SOD1 and TDP-43 have been implicated in MND (Hardiman et al., 2017), it remains unclear why certain CSN subpopulations preferentially degenerate in distinct MND subtypes. Identification and characterization of molecular controls that govern axonal development and connectivity of specific CSN subpopulations might provide insight regarding molecular mechanisms underlying preferential vulnerability of specific CSN subpopulations to degeneration.

Finally, our results suggest that Cbln1 might be a relevant molecular control for potential application in spinal cord repair and/or regeneration, and might elucidate broader organizing principles for establishing diverse connectivity by other neocortical projection neuron subtypes. Reactivating developmental controls to regulate CSN axon extension and sprouting might offer a promising approach to reestablish with some specificity damaged connectivity following spinal cord injury. More broadly, identification of molecular controls over development of anatomically and functionally diverse CSN subpopulations might elucidate fundamental principles of evolutionary diversification within originally more homogeneous neuronal populations and circuitry, while also offering potentially novel avenues for regeneration and/or repair of diseased and/or damaged neocortical or other nervous system circuitry.
